# Induction and suppression of tick cell antiviral RNAi responses by tick-borne flaviviruses

**DOI:** 10.1093/nar/gku657

**Published:** 2014-07-22

**Authors:** Esther Schnettler, Hana Tykalová, Mick Watson, Mayuri Sharma, Mark G. Sterken, Darren J. Obbard, Samuel H. Lewis, Melanie McFarlane, Lesley Bell-Sakyi, Gerald Barry, Sabine Weisheit, Sonja M. Best, Richard J. Kuhn, Gorben P. Pijlman, Margo E. Chase-Topping, Ernest A. Gould, Libor Grubhoffer, John K. Fazakerley, Alain Kohl

**Affiliations:** 1 MRC - University of Glasgow Centre for Virus Research, Glasgow G11 5JR, UK; 2 The Roslin Institute and Royal (Dick) School of Veterinary Studies, University of Edinburgh, Easter Bush, Midlothian EH25 9RG, UK; 3 Faculty of Science, University of South Bohemia and Biology Centre, Institute of Parasitology, Czech Academy of Sciences, 37005 České Budějovice (Budweis), Czech Republic; 4 Markey Centre for Structural Biology, Department of Biological Sciences, Purdue University, West Lafayette IN 47907, USA; 5 Laboratory of Virology, Wageningen University, 6708 PB Wageningen, The Netherlands; 6 Institute of Evolutionary Biology and Centre for Infection Immunity and Evolution, University of Edinburgh, EH9 3JT, UK; 7 Innate Immunity and Pathogenesis Unit, Laboratory of Virology, Rocky Mountain Laboratories, Division of Intramural Research, National Institute of Allergy and Infectious Diseases, National Institutes of Health, Hamilton, MT 59840, USA; 8 Centre for Immunity, Infection and Evolution, University of Edinburgh, EH9 3JT, UK; 9 Unité des Virus Emergents, Faculté de Médicine Timone, 13385 Marseille Cedex 05, France; 10 Centre for Hydrology and Ecology, Maclean Building, Oxon OX10 8BB, UK

## Abstract

Arboviruses are transmitted by distantly related arthropod vectors such as mosquitoes (class *Insecta*) and ticks (class *Arachnida*). RNA interference (RNAi) is the major antiviral mechanism in arthropods against arboviruses. Unlike in mosquitoes, tick antiviral RNAi is not understood, although this information is important to compare arbovirus/host interactions in different classes of arbovirus vectos. Using an *Ixodes scapularis-*derived cell line, key Argonaute proteins involved in RNAi and the response against tick-borne Langat virus (*Flaviviridae*) replication were identified and phylogenetic relationships characterized. Analysis of small RNAs in infected cells showed the production of virus-derived small interfering RNAs (viRNAs), which are key molecules of the antiviral RNAi response. Importantly, viRNAs were longer (22 nucleotides) than those from other arbovirus vectors and mapped at highest frequency to the termini of the viral genome, as opposed to mosquito-borne flaviviruses. Moreover, tick-borne flaviviruses expressed subgenomic flavivirus RNAs that interfere with tick RNAi. Our results characterize the antiviral RNAi response in tick cells including phylogenetic analysis of genes encoding antiviral proteins, and viral interference with this pathway. This shows important differences in antiviral RNAi between the two major classes of arbovirus vectors, and our data broadens our understanding of arthropod antiviral RNAi.

## INTRODUCTION

Tick-borne arboviruses of the *Flaviviridae* family are highly relevant to public health (1). Much work on tick-borne arboviruses has been carried out with Langat virus (LGTV), isolated from *Ixodes granulatus* and *Haemaphysalis* ssp. ticks in Malaysia and Thailand and related to tick-borne encephalitis virus (TBEV) (1–4). Flaviviruses are positive-stranded RNA viruses. Viral proteins are encoded in a single open reading frame. The untranslated RNA regions (UTRs) at the genome termini regulate replication and translation (5–8).

Arbovirus infection of arthropod cells is characterized by little or no cytopathic effects (9). Studies of vector/arbovirus interactions suggests that this may be at least partly due to regulation of arbovirus replication by innate immune responses (10). Research on vector immune responses to arboviruses has focused on mosquitoes (11,12) despite the fact that many European/Asian arboviruses are tick-borne (13). Antiviral responses in mosquitoes rely on a small RNA-based mechanism called RNA interference (RNAi) (10,11). The exogenous small interfering (si)RNA pathway is especially important and can be induced by virus-derived long double-stranded (ds)RNA molecules generated during infection (either replication intermediates or secondary RNA structures) or dsRNA viral genome (10). In insects, dsRNA is targeted by the Dicer enzyme (Dcr-2) and cleaved into 21 nucleotide (nt) siRNAs, also known as viRNAs (10,11). In *Drosophila*, viRNAs are integrated into the Argonaute-2 protein (Ago-2) containing RNA-induced silencing complex, unwound and one strand of the viRNA is retained by Ago-2 to guide degradation of complementary (viral) RNA (14). Other Ago and Dcr proteins, i.e. Dcr-1 and Ago-1, are involved in the microRNA (miRNA) pathway (10–11,14).

Following treatment with gene-specific dsRNA or siRNAs, ticks and tick cell cultures can induce sequence-specific RNAi of endogenous genes (15) and restrict viral infections (16–18). Sequence analysis has also identified putative Ago and Dcr genes in the *I. scapularis* genome (19). However, it is not known if these are transcribed and involved in tick antiviral RNAi responses. All studied insect specific viruses and plant-infecting viruses have been shown to express RNA silencing suppressor (RSS) proteins which interfere with the RNAi response (20). No RSS proteins have been identified for arboviruses although evasion strategies have been suggested for the alphavirus Semliki Forest virus (SFV) (21), and the production of a subgenomic flavivirus RNA (sfRNA) interfering with the RNAi response was reported for mosquito-borne flaviviruses (22).

In this study, we identify and characterize key RNAi players of the Ago family that interfere with LGTV replication and describe characteristics of viRNAs in tick vector cells, which are different to viRNAs in mosquitoes. We also demonstrate that the recently described RSS activity of mosquito-borne flavivirus sfRNA can be broadened to tick-borne LGTV and TBEV sfRNA. The results imply that the antiviral RNAi system in ticks is more complex and has important differences to that of mosquitoes.

## MATERIALS AND METHODS

### Viruses and plasmids

The LGTV replicon (E5repRluc2B/3) was derived from the infectious cDNA of LGTV E5 (4). Modifications in the LGTV replicon were based on the previously described replicon construct for TBEV Neudoerfl strain (23). This construct encodes the first 17 residues of capsid, followed by the Rluc gene, the last 27 residues of the envelope and all non-structural proteins, as described in Supplementary Data. For infections of tick cells, LGTV strain TP21 was used.

Invertebrate expression vectors, pIZ-Fluc, pAcIE1-Rluc and pIB-MBP-HDVr have been described previously (22). The 3′UTRs of LGTV and TBEV were amplified by polymerase chain reaction (PCR) using, respectively, E5repRluc2B/3 or pTNd/ΔME (24) as templates. Invertebrate expression plasmids were obtained by fusing the 3′ terminus to the HDVr sequence from a WNV 3′UTR expression construct (22) using PCR. The resulting products were cloned into pDonor207 and pIB-GW plasmids (Invitrogen) using Gateway technology.

### Luciferase assays

Luciferase activities were determined using a Dual Luciferase assay kit (Promega) in a GloMax multi-luminometer following cell lysis in Passive Lysis Buffer.

### Cell culture, transfection and infection

BHK-21 cells were grown in GMEM at 37°C as previously described (25). Cells (3 × 10^5^/well) were seeded in a 6-well plate prior to transfection with Lipofectamine2000 (Invitrogen) according to the manufacture's protocol. The *I. scapularis*-derived IDE8 cells were grown in L-15B medium (26) at 32°C in ambient air as previously described (27). Cells (6.5 × 10^5^/well) were seeded in 24-well plates prior to transfection. Transient RNAi suppression assays were performed by transfecting 200 ng pIZ-Fluc, 300 ng pAcIE1-Rluc and 500 ng pIB-MBP-HDVr, TBEV 3′UTR or LGTV 3′UTR into IDE8 cells using Genejammer (Agilent) following the manufacturer's instructions. Silencing of reporter genes was induced at 24 h post-transfection (hpt) through addition of 280 ng dsRNA to the cell culture medium; luciferase was measured 48 hpt.

In case of studies involving replicon, putative RNAi genes were silenced by the addition of 300 ng dsRNA to cell culture medium at 6 and 30 h post-seeding (hps). Then, capped *in vitro-*transcribed E5repRluc2B/3 was transfected 48 hps using Lipofectamine2000 according to the manufacturer's instructions. Luciferase expression was measured 24 hpt.

For infection assays, target genes were first silenced by transfection of 100 ng dsRNA using Lipofectamine2000, followed by LGTV TP21 infection at 24 hpt at a multiplicity of infection (MOI) of 0.1. RNA was isolated at 48 h post infection (hpi) by Trizol.

### Statistical analysis

The relative luciferase expression (RL) was calculated as:

\begin{equation*} {\rm RL}_i = {\rm I}_{{\rm Fluc},i} /{\rm I}_{{\rm Rluc},i} \end{equation*}

Where I is the measured intensity and *i* is the sample. To cancel out construct specific effects, values under treatment (for example co-transfected with dsFFluc) were normalized against the same construct that was treated with a negative control (in this example dseGFP). Thus:

\begin{equation*} {\rm NRL}_{\rm x} = {\rm RL}_{i,{\rm treated}} /{\rm RL}_{i,{\rm neg}{\rm .control}} \end{equation*}

Experiments were performed in duplicate or in triplicate and repeated independently at least three times. The independent experiments were averaged:

\begin{equation*} \overline {{\rm NRL}} = \sum\limits_x^n {\frac{{{\rm NRL}_x }}{n}} \end{equation*}

Where *x* is the *x*th experiment and *n* is the total number of experiments.

The significances were calculated using custom-written scripts in R (www.r-project.org). In case of pairwise testing a two-sample independent *t*-test was performed, as provided by R.

Multiple testing was done by applying Tukey's HSD (also known as Tukey's range test), the *q*-value was calculated and compared to the indexed q in the studentized range distribution available in R. Significant differences (*P* ≤ 0.05) are indicated in the graphs with an *.

### Small RNA isolation and deep sequencing analysis

1.5 × 10^6^ cells per tube were either transfected with 1 μg of eGFP-derived dsRNA, capped *in vitro-*transcribed E5repRluc2B/3 RNA, infected with LGTV TP21 (MOI 10) or untreated. At 48 hpt or 72 hpi, RNA was isolated using 1 ml Trizol (Invitrogen) per tube, small RNAs of 18–30 nt were sequenced and analyzed using viRome as previously described (28,29). Small RNA data was submitted to the European Nucleotide Archive (accession number ERP006219).

### Reverse transcription and PCR

RNA was isolated by Trizol, following the manufacturer's protocol. Total RNA (500 ng for untreated/dsRNA-treated cells as well as knockdowns followed by LGTV infection or 5 μg LGTV antigenome detection) was reverse transcribed with Superscript III (Invitrogen) and using either oligo dT primers (knockdowns), an antigenome specific primer (LGTV antigenome detection) or random hexamers (LGTV infection) following the manufacturer's instructions. For the detection and amplification of Ago and Dcr transcripts, PCR was carried out using 2 μl of the cDNA reaction with corresponding primers (Table 1). The eGFP-derived PCR product was produced using eGFP-C1 (Clontech) as template. In case of LGTV antigenome detection, two rounds of PCR were performed using LGTV specific primers. PCR products were gel-purified, cloned into the pJet blunt1.2 vector (Fermentas) and sequenced.

**Table 1.** List of primer sequences used

Gene	Upstream/downstream primer sequences (5′-3′)
Ago-68	*gtaatacgactcactataggg*CGAGACTTTCAGAGCGTG/ *gtaatacgactcactataggg* GTTGGTGTACTTCGCCAT
Ago-30	*gtaatacgactcactataggg*ACATACGAGCACTGACGG/ *gtaatacgactcactataggg*TGGTGCAACATTTTATCGA
Ago-30–2	*gtaatacgactcactataggg*GAACGCCAAAAAGATCCCA/ *gtaatacgactcactataggg*CCGGTACCATCCTCATTTCT
Ago-16	*gtaatacgactcactataggg*AAGATCACGAGGGTATCGGTAGT/ *gtaatacgactcactataggg*ACTTTTCTGCACCACGTCTTG
Ago-16-2 (RT-PCR detection)	*gtaatacgactcactataggg*CGTTATGAAGGGTGATCAGAAG/ *gtaatacgactcactataggg*GACTGGTACTGATTCTCCCA
Ago-96	*gtaatacgactcactataggg*ATGCCTGCTCGGACATCTAC/ *gtaata cgactcactataggg*TCGAGTGAACGTCCAAATTCT
Ago-78	*gtaatacgactcactataggg*GAGGTGAAGCGTGTGGGG/ *gtaatacga ctcactataggg*GATGGAAGGCTTCTTGTTGTC
Dcr-90	*gtaatacgactcactataggg*ATCCTCAAGGAGTACAAGCC/ *gtaata cgactcactataggg*ACAGAGCATTAGGGTCGTC
Dcr-98	*gtaatacgactcactataggg*ATCCCGTCTTTCCCGATCTT/ *gtaatacgactcactataggg* TGCATCACAGGTGCCAGG
eGFP	*gtaatacgactcactataggg*GGCGTGCAGTGCTTCAGCCGC/ *gtaatacgactcactataggg* GTGGTTGTCGGGCAGCAGCAC
Firefly luciferase	*gtaatacgactcactataggg*ATGGAAGCAGCCAAAAAC/ *gtaatacgactcactataggg* TTACACGGCGATCTTTCC
LGTV antigenome RT-PCR	aattccacccatgaaatgtac
LGTV NS5 (QT-PCR)	acccaagactgctacgtgtggaaa/tgaggaagtaaagggccttgctga

T7 promoter region is indicated in italics.

LGTV RNA was determined by QRT-PCR with NS5 specific primers using the Fast SYBR Green PCR Master Mix (Life Technologies) according to manufacturer's instructions. Previously described actin primers were used as housekeeping genes (16).

### *In vitro* transcription and dsRNA production

E5repRluc2B/3 was linearized by EcoRV and *in vitro-*transcribed using a SP6 Megascript kit (Ambion) in the presence of cap analogue according to the manufacturer's protocol. dsRNA was produced with the RNAi Megascript kit (Ambion) from PCR products flanked by T7 promoter sequences.

### Phylogenetic analysis

To place the *Ixodes* sequences within gene trees, representative sequences were downloaded from Genbank (see Supplementary Figure S5 for sequence identifiers) for selected arthropods (waterfleas, copepods, lice, ticks, centipedes, flies, butterflies, beetles and wasps) and deuterostomes (sea squirt, human, chicken and zebrafish) that have sufficient complete genomes and/or transcriptomes. Ago and Piwi were aligned with translational MAFFT (30) and poorly-aligned regions were removed manually, resulting in an aligned matrix of 2349 positions for Ago and 2241 positions for Piwi. Due to a higher level of sequence divergence and higher proporation of incomplete orthologus sequences, Dicer was aligned under a codon model using PRANK (31), and then GBLOCKs (32) was used to exclude regions of poor alignment, resulting in an aligned matrix of 810 positions. Gene trees were inferred with MrBayes (33) using unlinked General Time Reversible models with Gamma-distributed rate variation for each of the three codon positions. Two parallel MCMC chains of >25 million steps were run for each tree (sampling every 1000 steps) and the first 25% of steps were discarded as burn-in. Stationarity was inferred by comparing parallel runs and inspection of the chains for each parameter: Potential Scale Reduction Factors approached 1.000, the variation in split frequencies was <0.01, and effective sample sizes were >1000 for all parameters. The trees presented in Figure 2 and Supplementary Figure S5 are maximum clade credibility trees, with branch lengths proportional to the number of substitutions. Only partitions with >90% Bayesian Posterior support are labeled.

**Figure 1.**
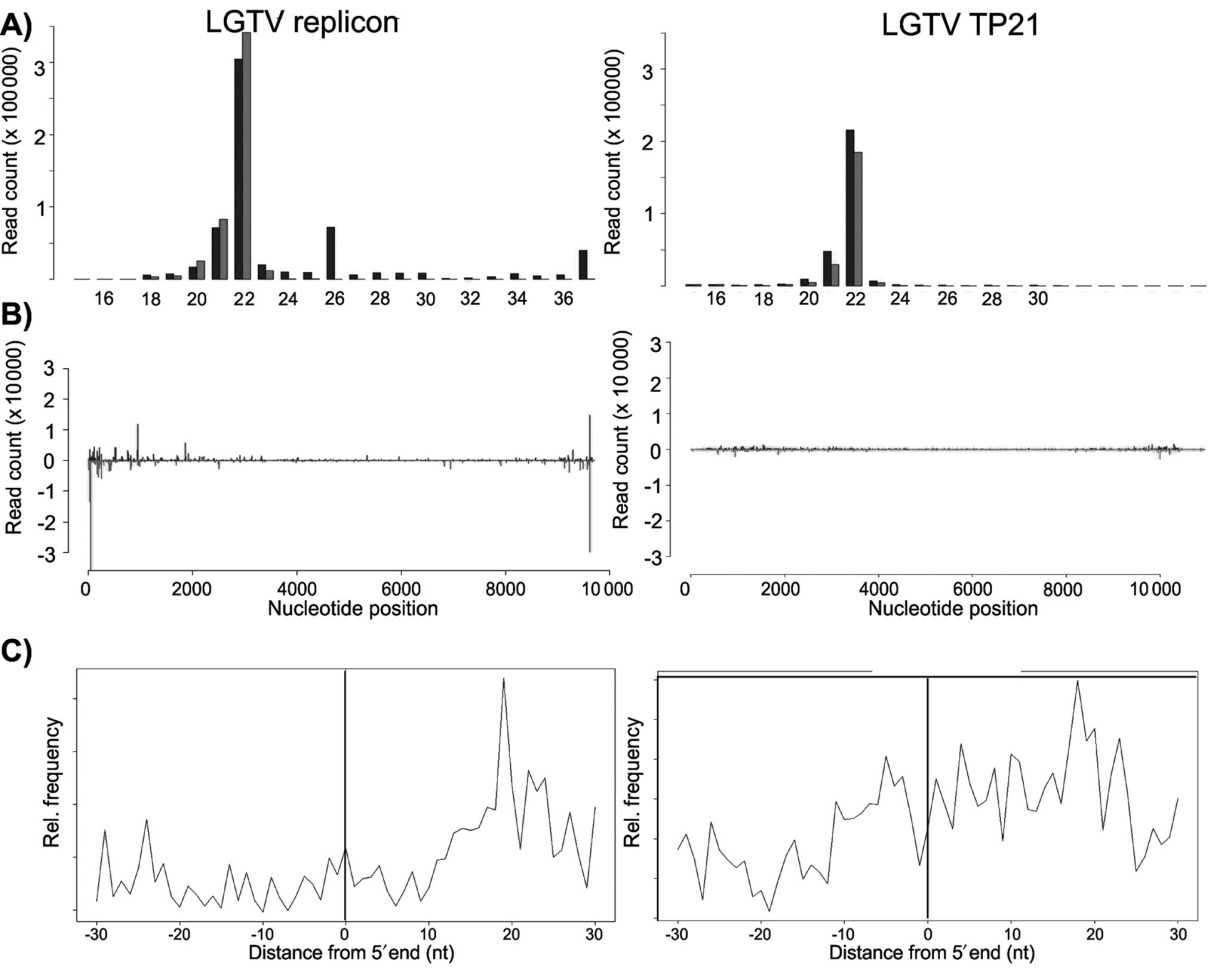
Characterization of exogenous-derived small RNAs in IDE8 cells. (**A**) Size distribution of small RNA molecules mapping either to LGTV E5repRluc2B/3 replicon (left panel) at 48 hpt or LGTV TP21 (right panel) at 72 hpi in IDE8 cells. (**B**) Frequency distribution of 22 nt small RNA molecules mapped to the E5repRluc 2B/3 replicon (5′UTR to 3′UTR) (left panel) or LGTV TP21 (right panel). The y-axis shows the frequency of the 22 nt siRNAs mapping to the corresponding nucleotide position in the x-axis. Positive numbers and dark gray peaks represent the frequency of siRNAs mapping to the genome (in 5′-3′ orientation) and light gray peaks/negative numbers to the antigenome (in 3′-5′ orientation). See also Supplementary Figure S2. (**C**) Frequency map of 22 nt small RNAs mapping to the opposite strand of the LGTV replicon (left panel) or LGTV TP21 (right panel).

**Figure 2.**
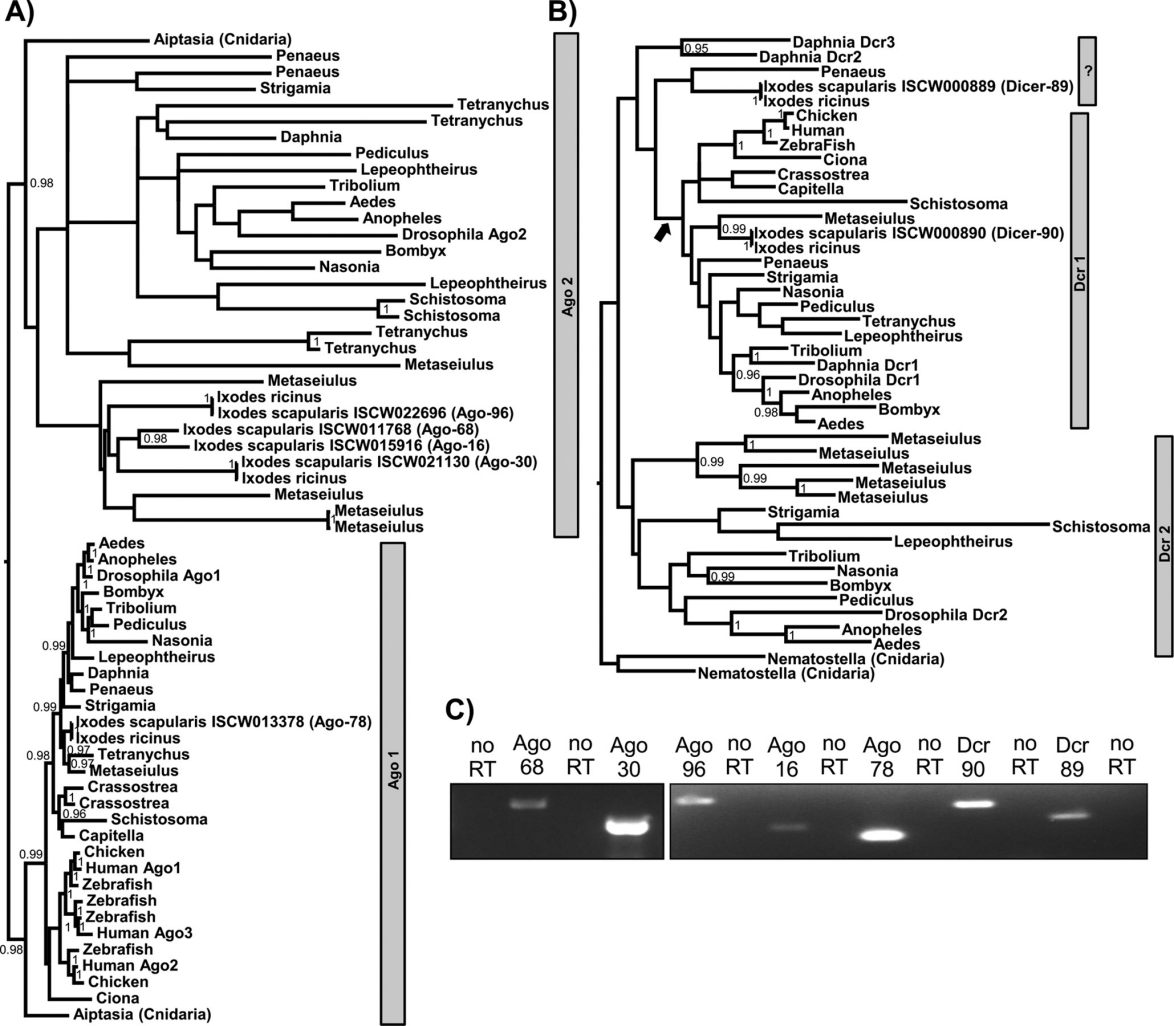
Analysis of Ago and Dcr protein-encoding genes in the *Ixodes scapularis* genome. (**A**) and (**B**) are gene trees for metazoan Ago-subfamily genes **(A)**and Dcr genes **(B)** respectively, constructed using a Bayesian approach under a GTR model (nodes are labeled if they receive >90% support; see Materials and Methods). Trees are unrooted, but presented as if the root fell between the two Cnidarian homologs. (**C**) Detection of transcripts encoding Ago and Dcr proteins in IDE8 cells by RT-PCR. RNA not treated with reverse transcriptase was used as control during the PCR reaction (no RT). See also Supplementary Figure S5.

### RNA structure predictions

Consensus RNA structures were predicted using the LocARNA web server (Vienna RNA web server 1.8.2) (34) with standard settings. Pseudoknots were identified manually. Thermodynamic stability was calculated by folding an individual sequence with RNAfold (Vienna RNA web server 1.8.2), using a secondary structure constraint and standard settings.

### Northern blot analysis

Northern blot analysis was performed by loading 4.5 μg or 3 μg of total RNA of BHK-21 or tick cells, respectively, on a 1.5% agarose-2% formaldehyde MOPS gel and transferred to a nitrocellulose (Hybond-N+, GE Healthcare) membrane using ‘top down’ blotting with 20xSSC as transfer buffer. Transferred RNA was UV-crosslinked for 2 min. Hybridization was performed for 2 h in HybPerfect buffer (Sigma) at 63°C using DIG-labeled PCR product as probe (TBEV 3′UTR or LGTV 3′ UTR). Membranes were washed twice with 2xSSC + 0.1% SDS for 5 min, twice with 0.2xSSC + 0.1% SDS for 20 min at 63°C and DIG was detected using an anti-DIG antibody as described previously (35).

## RESULTS

### I. *scapularis-*derived-IDE8 cells mount RNAi responses against LGTV and TBEV

An uncharacterized RNAi response was shown to restrict mosquito-borne arbovirus infections in *I. scapularis*-derived ISE6 and IDE8 cells (16–18). It is not known if RNAi is induced in tick cells following infection with tick-borne arboviruses. Production of viRNAs is an indicator of an antiviral RNAi response. An LGTV E5 strain replicon encoding the *Renilla* luciferase (Rluc) gene as a reporter (E5repRluc2B/3) was constructed to investigate antiviral RNAi in IDE8 cells (Supplementary Figure S1A). The ability to successfully transfect E5repRluc2B/3 RNA into IDE8 cells (77%) was determined, using either fluorescently labeled replicon RNA or immune-fluorescence detection of NS3, respectively (Supplementary Figure S6A). Following transfection with E5repRluc2B/3 RNA, IDE8 cells were lysed and Rluc expression determined at 24, 48, 72, 96 and 120 hpt. Expression was observed 24 hpt then decreased (Supplementary Figure S1C). Replication was verified by detection of LGTV antigenome (Supplementary Figure S1B). These results suggest that the LGTV replicon is inhibited by an induced antiviral response in IDE8 cells.

Previous work has documented the production of viRNAs in ISE6 cells; however, the sequences and their distribution on the virus genome are not known (17,18). The production of LGTV-specific viRNAs in IDE8 cells was therefore analyzed. At 48 hpt, total RNA was isolated and small RNAs sequenced; frequencies and LGTV genome location of small RNAs were determined (Table 2). 7.1% of the small RNA sequences mapped to the LGTV replicon sequence. viRNAs were predominantly 22 nt in length (59.6%) and mapped with similar frequency to the genome and antigenome (Figure 1A, left panel). viRNAs were scattered along the LGTV replicon genome/antigenome with variable frequency into hot spots/cold spots (21) (Figure 1B, left panel). The 5′ and 3′UTRs generated the highest viRNA frequencies (Figure 1B, left panel). Comparing the base composition of 22 nt viRNAs of hot spots versus cold spots showed a substantial bias away from G toward A at the 5′ end (*P* < 0.0001, Fishers exact test [FET]) and a bias away from A at the 3′ end (*P* < 0.0001, FET). Bias at other positions was found but none was particularly striking (Supplementary Figure S2A). The 5′ ends of the complementary LGTV specific 22 nt RNAs were most frequently separated by 20 nt (Figure 1C, left panel) suggesting generation from dsRNA of 20 nt with 2 nt overhangs. Experiments performed with a previously described TBEV replicon (23) (Supplementary Figure S2C) showed similar results regarding the predominance of 22 nt viRNAs and 5.9% of total small RNAs mapping to the TBEV replicon (Table 2), with similar frequency to the genome and antigenome. The 22 nt small RNAs mapping to TBEV, are scattered along the genome/-antigenome. Again the highest frequency of viRNAs was generated from the 5′ and 3′ UTRs (Supplementary Figure S2D). Experiments with IDE8 cells infected with LGTV TP21 showed the production of virus specific small RNAs sharing several of the characteristics of LGTV replicon-derived viRNAs, although at a lower overall frequency (0.27% for virus and 7.12% for replicon (Table 2)). The majority of viRNAs were 22 nts in length, most frequently separated by 20 nts and the highest viRNA frequencies were generated from and around the 5′ and 3′ ends of the viral genome/antigenome (Figure 1, right panels and Table 2).

**Table 2.** Number of small RNA reads

	Langat virus replicon	St. Croix River virus	Langat virus TP21	TBEV replicon	TBEV NS5 GAA replicon
Genome/coding strand reads	719782	1462846	294390	3906753	3153338
Anti-genome/coding strand reads	553286	1242195	227753	3606509	2921677
Total viral reads	1273068	2705041	522143	7513262	6075015
Reads in total	17875799	18806256	190908946	127799016	127066875

Indicated are read numbers of small RNAs mapping to the genomes and antigenomes of St. Croix River virus, Langat virus replicon, Langat virus TP21 and TBEV replicons.

### The length of small RNAs in IDE8 cells is a host property

Recent studies have shown that insect viRNAs are generally 21 nt in length, in contrast to nematode *Caenorhabditis elegans* viRNAs of predominantly 22 or 23 nt depending on the virus (21,28,36–47). To determine whether generation of 22 nt as the dominant viRNA length was a property of the cells or the virus, an eGFP-derived dsRNA was transfected into IDE8 cells and small RNAs analyzed. Again, 22 nt was the dominant length (Supplementary Figure S2E) and small RNAs mapped in hot/cold spots along the whole eGFP sequence and its complement (Supplementary Figure S2E).

We also analyzed viRNAs targeting the dsRNA orbivirus St. Croix River virus (SCRV) (48,49), which persistently infects IDE8 cells (Table 2). Again, the majority of SCRV viRNAs were 22 nt, with similar frequencies being detected on the (+) and the (–) strand (Supplementary Figure S3).

To determine the properties of endogenous small RNA molecules such as miRNAs, endogenous siRNAs and PIWI-interacting (pi)RNAs (10) in IDE8 cells, the small RNA profiles from uninfected and treated (eGFP dsRNA and LGTV replicon) IDE8 cells were analyzed. Small RNAs mapping to the *I. scapularis* genome (https://www.vectorbase.org) had a predominant length of 22 nt (44.4%) in all samples, with slightly higher frequencies for the sense orientation. Moreover, a class of small RNA molecules of 27 to 29 nt was identified with a peak at 28 nt (27 nt: 6.7%, 28 nt: 10.1% and 29 nt: 5%) as strongly represented as 21 nt small RNAs (12.5%) (Supplementary Figure S4). This indicates that 22 nt is the dominant length of small RNAs (endogenous or viral) in IDE8 cells.

### Identification of Dcr and Ago proteins involved in antiviral RNAi in tick cells

Ago-2 and Dcr-2 proteins are key effectors in the insect antiviral RNAi pathway (10,50). Dcr-1 and Ago-1 are known to be important for the insect miRNA pathway (10,14). Previous sequence analysis has shown that the *I. scapularis* genome contains at least one putative Dcr gene, Dcr-89 (ISCW000889) and two putative Ago subfamily genes; Ago-68 (ISCW011768), Ago-30 (ISCW0021130) (19). In the present study, Basic Local Alignment Search Tool (BLAST) similarity searches with Dcr (Dcr-1 and Dcr-2) and Ago subfamily genes (Ago-1 and Ago-2) of *Drosophila melanogaster* and *Aedes aegypti* were performed to identify further putative homologs in the *I. scapularis* genome. Three additional putative Ago subfamily genes; Ago-96 (ISCW022696), Ago-16 (ISCW015916), Ago-78 (ISCW013378) and another putative Dcr gene, Dcr-90 (ISCW0008890) were identified.

To understand the function of *Ixodes* Ago and Dcr proteins within the wider context of their evolution, gene trees were constructed using a Bayesian approach (Figure 2A and B; Supplementary Figure S5). The last common ancestor of each of these gene families probably pre-dates the origin of the animals (51), so that saturation and long-branch artifacts make reliable tree inference extremely challenging. Rooting the Ago tree between the two cnidarian paralogs identified two well-supported clades: the slowly evolving clade homologous to drosophila Ago-1 (miRNA pathway) and the rapidly evolving clade homologous to drosophila Ago-2 (siRNA pathway). The Ago-1 clade exactly mirrors the known phylogeny of the species, and clearly identifies Ixodes Ago-78 as an ortholog of drosophila Ago-1. The other four *Ixodes* Ago (-96, -68, -16, -30) then appear as more recent Ago-2 paralogs that have evolved since the last common ancestor of *Arachnida* and Pancrustacea, although a lack of support within this clade makes it hard to draw conclusions beyond this. The Dcr gene-tree also lacks support, and when similarly rooted using the cnidarian paralogs results in a pattern that is hard to interpret. With this rooting, *Ixodes* Dcr-90 clusters with other arachnid Dicers basal to an arthropod clade that includes drosophila Dcr-1, suggesting that Dcr-90 is a Dcr-1 homologue. However, the basal position of *Ixodes* Dcr-89 and the remaining crustacean Dicers is difficult to reconcile with the known organismal phylogeny. If the divergent Cnidarian outgroup is excluded, then an alternative rooting immediately basal to the deuterostome/arthropod Dcr-1 clade (marked by a black arrow in Figure 2B) would place Dcr-89 and the remaining Crustacean Dcrs as the most basally-branching arthropod Dcr-2, consistent with the species phylogeny and suggesting it is homologous to drosophila Dcr-2. Transcription of putative Ago and Dcr genes was verified in IDE8 cells (Figure 2C).

**Figure 3.**
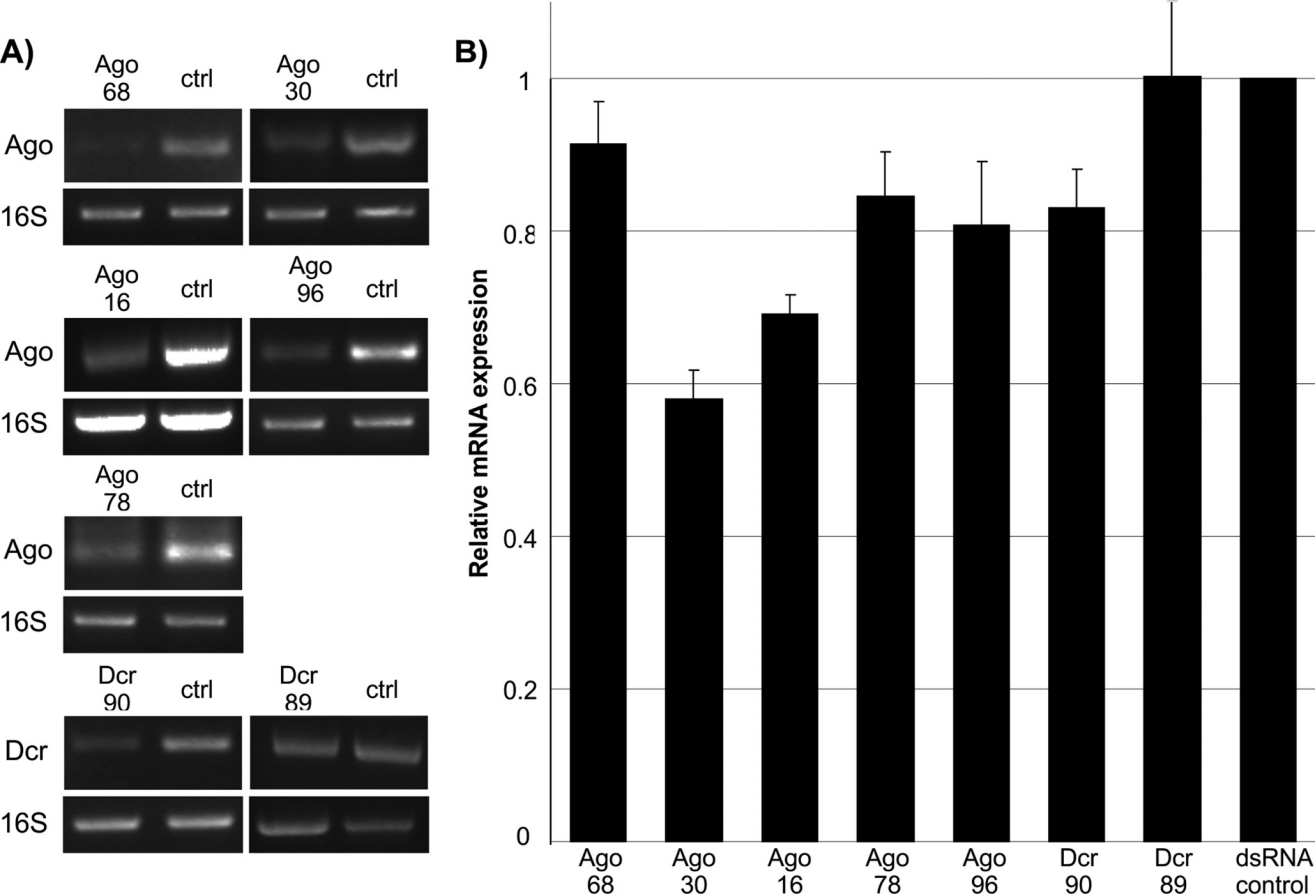
Knockdown of transcripts encoding Ago or Dcr proteins. (**A**) dsRNA-based silencing of Ago and Dcr encoding transcripts in IDE8 cells. Transcripts were detected by RT-PCR using gene-specific primers. A PCR product of 16S ribosomal RNA was used as housekeeping gene and eGFP specific dsRNA treated cells as control (crtl). (**B**) mRNA knockdowns quantification by Image J software, using 16S as control. The relative mean (normalized to eGFP-dsRNA controls) with standard error is shown for at least 10 repeats.

In order to investigate mediators of antiviral activity in IDE8 cells, transcripts of individual Dcr or Ago genes were knocked down by RNAi as previously described (16) and the effect on the LGTV replicon determined. Efficiency of knockdown/silencing of cells treated with dsRNA specific for Ago (Ago-68, Ago-30, Ago-16, Ago-96 and Ago-78) and Dcr (Dcr-90 and Dcr-89) genes was determined by semi-quantitative RT-PCR and quantified in relation to control dsRNA using 16S as loading control (Figure 3). Cells treated with dsRNA against Ago-68, Ago-30, Ago-96, Ago-16, Ago-78 or Dcr-90 showed reduction in target transcript levels (9–40%). No significant reduction of Dcr-89 transcript was observed, due to a high variability between samples (Figure 3).

**Figure 4.**
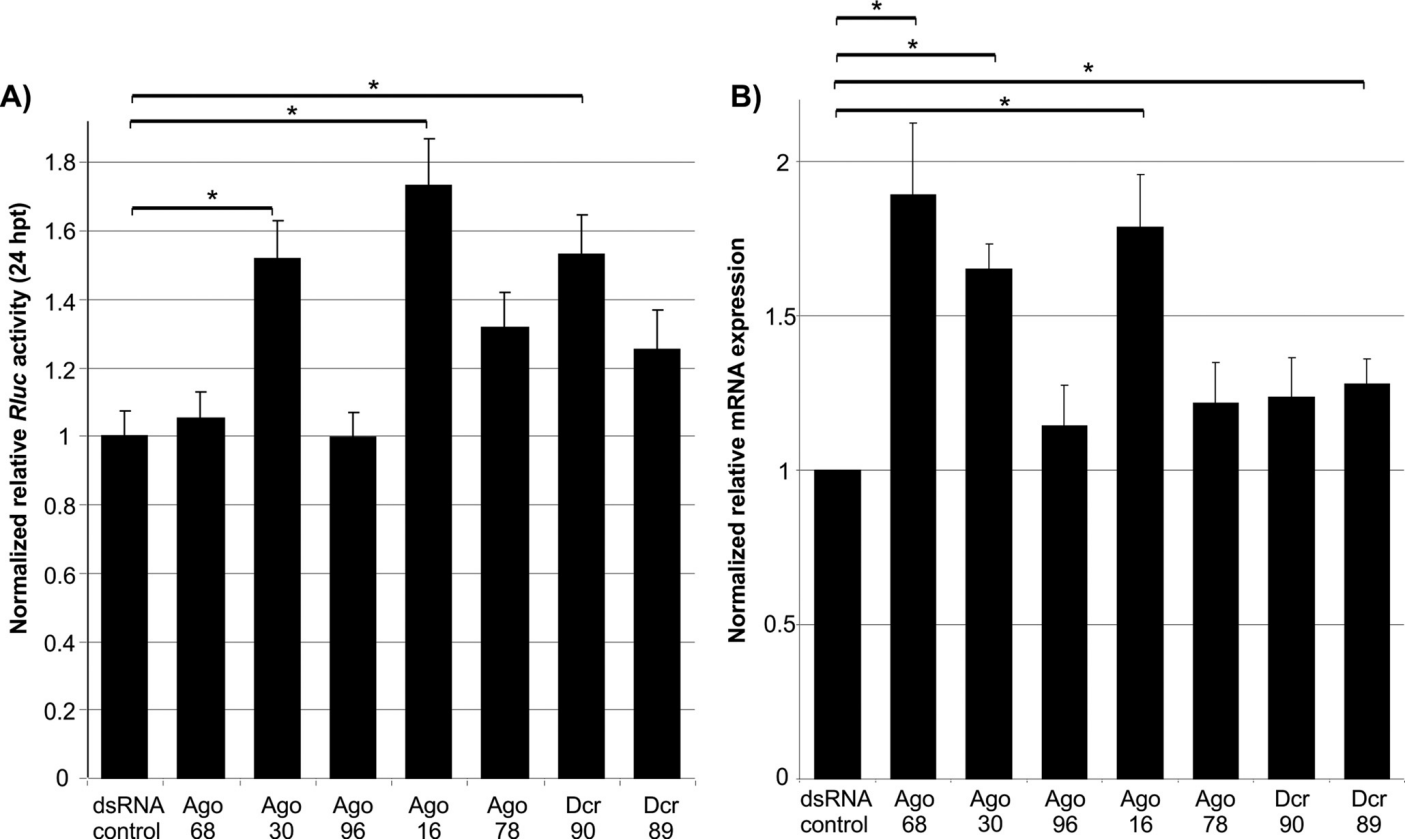
Effects of Ago and Dcr knockdowns on LGTV replication. (**A**) Ago or Dcr silenced cells were transfected with capped *in vitro*- transcribed LGTV E5repRluc2B/3 replicon RNA, and Rluc activity was determined at 24 hpt. The mean with standard error is shown for three independent experiments performed in duplicate (two experiments)/triplicate (one experiment). The data were normalized to cells treated with eGFP-specific control dsRNA. * indicate significance by Tukey's HSD (*P* ≤ 0.05). (**B**) Silenced cells were infected with Langat virus (MOI 0.1) and viral RNA was determined by QRT-PCR at 48 hpi, using actin as housekeeping gene internal standard. The mean with standard error is shown for three independent experiments performed in triplicate. The data were normalized to cells treated with eGFP-specific control dsRNA. * indicate significance by Student *t*-test (*P* ≤ 0.05).

Following successful individual knockdowns of most putative RNAi genes, the experiment was repeated, LGTV replicon RNA was transfected into silenced IDE8 cells and replicon-mediated Rluc activities determined. Significant increases in replicon Rluc activity were observed in IDE8 cells treated with dsRNA specific for Ago-30, Ago-16 and Dcr-90, compared to control dsRNA (Figure 4A). No significant increase of Rluc was observed following Ago-68, -78, -96 and Dcr-89 knockdowns. To ensure that the observed effect was not due to off target effects, the Ago-30 knockdown was repeated with an additional Ago-30 specific dsRNA molecule (Ago30–2); this resulted in a similar increase of luciferase activity thus confirming previous results (Supplementary Figure S6B). Similar experiments were also performed with silenced IDE8 cells and the effect on LGTV infection (MOI 0.1) at 48 hpi was determined by QRT-PCR. Significant increases in LGTV RNA levels were observed in cells treated with dsRNA specific for Ago-68, -30, -16 and Dcr-89, although Dcr-89 resulted only in a small increase (Figure 4B).

Targeting of the same cells by dsRNA and LGTV replicon or LGTV infection was established, using fluorescently labeled dsRNA and immunostaining of LGTV NS3 or E protein (Supplementary Figure S6A). In summary, tick Ago-30 and Ago-16 mediate antiviral activity against both LGTV and its replicon.

### Tick-borne subgenomic flavivirus (sf)RNA interferes with antiviral RNAi

sfRNA is derived from the flavivirus 3′UTR, produced in vertebrate and invertebrate cells by mosquito and tick borne-flaviviruses and contains a complex RNA structure (52–54). West Nile virus (WNV) and dengue virus (DENV) sfRNAs both interfere with RNAi (22).

Production of sfRNA and suppression of RNAi by both LGTV and TBEV was investigated. The 3′UTRs of flaviviruses share common characteristics in their RNA architecture (55). It has been demonstrated that mosquito-borne flaviviruses share an RNA stem loop structure (called SL II) toward the 5′ end of the 3′UTR which has similarities to SL IV of the 3′UTR and is important for sfRNA production (52–54). RNA folding predictions of the 3′UTR of tick-borne flaviviruses showed RNA structures with folds highly similar to SL II and SL IV (respectively named SL 2 and SL 1 in the tick-borne viruses) for most tick-borne flaviviruses, despite sequence differences to mosquito-borne flavivirus 3′UTRs (Supplementary Figure S7).

**Figure 5.**
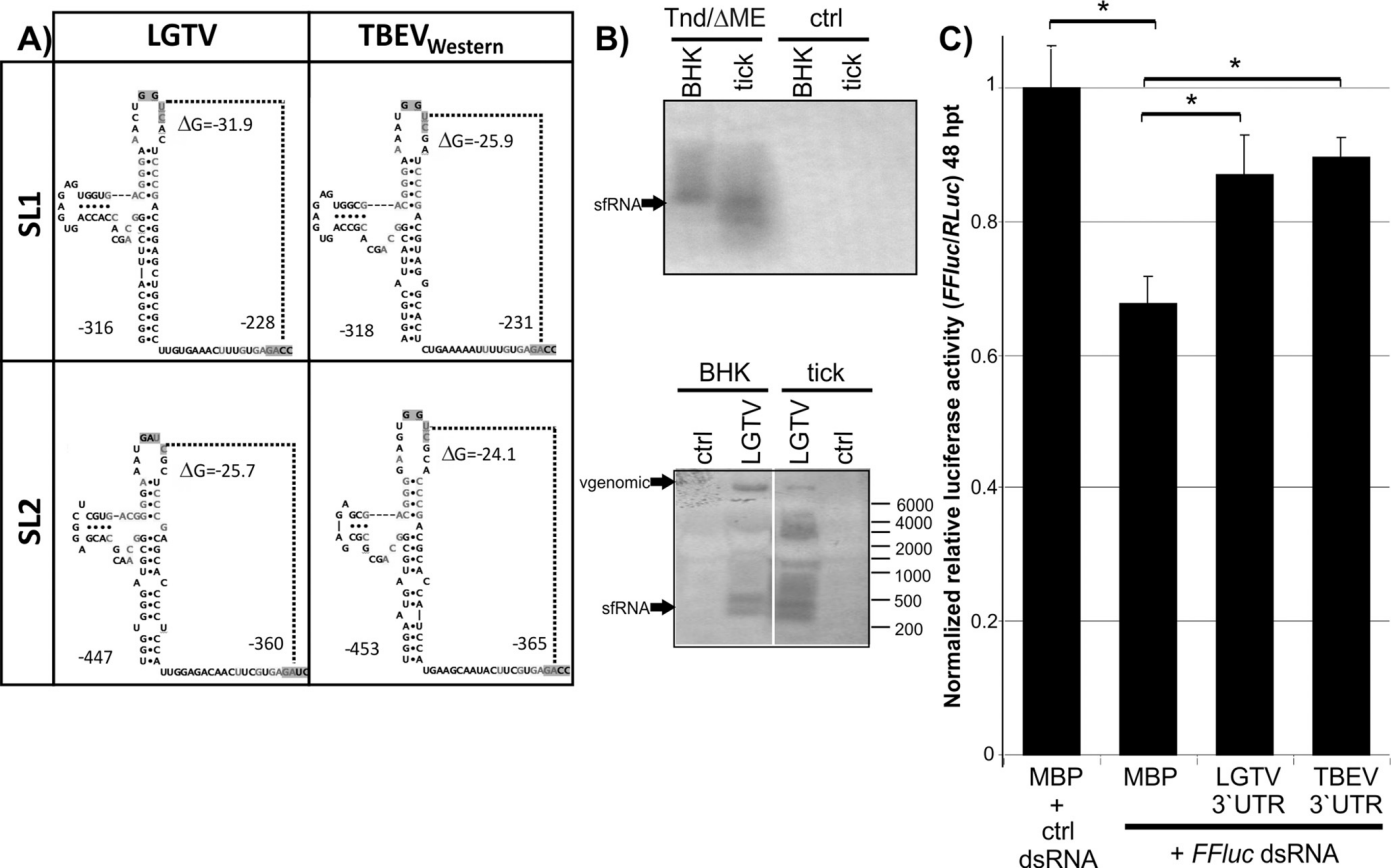
Analysis of subgenomic flavivirus (sf)RNA in the 3′UTR of tick-borne flaviviruses. (**A**) Structure model of SL 2 and SL 1 RNA stem loop structures of TBEV and LGTV. (**B**) Expression of TBEV (TND/ΔME) (top) and LGTV (bottom) sfRNA in replicon (top), non-transfected (control CTRL) or infected cells (bottom) was detected by northern blot analysis with 3′UTR specific DIG-PCR probes. (**C**) The effect of sfRNA on RNAi in IDE8 cells was determined by co-transfection of *FFluc*, Rluc and expression constructs for MBP-HdVr (MBP), LGTV 3′UTR or TBEV 3′UTR. Silencing was induced 24 hpt following addition of dsRNA to the culture medium. At 48 hpt, relative luciferase activity (*FFluc*/Rluc) was determined and normalized to cells treated with eGFP specific (ctrl) dsRNA. The luciferase expression level measured with MBP-HdVr was set at 1.0. The mean with standard error is shown for three independent experiments performed in duplicate (one experiment)/triplicate (two experiments). * indicate significance by Tukey's HSD (*P* ≤ 0.05). See also Supplementary Figure S7.

To determine if the predicted LGTV and TBEV RNA stem loop structures (Figure 5A and Supplementary Figure S7) give rise to sfRNAs, vertebrate and tick cells were infected with LGTV (56) or transfected with TBEV replicon (24). Northern blot analysis detected TBEV and LGTV RNA at the expected size of ∼0.4 kb (predicted SL 2, LGTV: −447 nt; TBEV: −453 nt) (Figure 5A and B). In addition, similar to WNV a lower band was observed. This may be due to the presence and characteristics of two SL structures [SL 1 and 2]. Moreover, there are differences between arthropod and vertebrate cells (Figure 5B) (52,53).

The RSS activity of these sfRNAs was investigated next, after establishing successful plasmid transfections in IDE8 cells (Supplementary Figure S6A). IDE8 cells were co-transfected with plasmids encoding Firefly luciferase (*FFluc*; reporter gene), Rluc (internal control), and plasmids expressing LGTV or TBEV 3′UTRs. Maltose binding protein (MBP) sequence fused to the hepatitis delta virus ribozyme (HDVr) was used as negative control RNA as the 3′UTRs plasmids also contain an HDVr. Subsequently, silencing was induced by either *FFluc*-specific (ds*FFluc*) or control (eGFP) dsRNA and luciferase activity determined. Reduced silencing was observed in cells expressing 3′UTR constructs compared to MBP-HDVr (Figure 5C). These results indicate that the 3′UTRs of LGTV and TBEV are able to interfere with the tick siRNA pathway.

## DISCUSSION

RNAi is known to be a major defense mechanism against arboviruses in mosquitoes (10,11). Much less is known about ticks. Here, we investigated the antiviral RNAi response in *I. scapularis*-derived cells and viral counter-defense strategies. Our analysis reveals tick Ago and Dcr genes additional to those previously described (19). A significant gene expansion in the Ago subfamily has occurred in arachnids, compared to insects such as *D. melanogaster* and *A. aegypti*. Our results characterize key differences between *Ixodes* and mosquito RNAi responses. The antiviral activity of Ago-30, Ago-16 and Ago-68 (in case of viral infection) is in line with previous reports showing that mosquito/fly Ago-2 is involved in the antiviral RNAi response and phylogenetic analysis indicates that *Ixodes* Ago-30, Ago16 and Ago-68 are homologous to Ago-2 of insects (57,58). The expansion of putative Ago-2 paralogs in arachnids is different from other arthropods, which generally have one, or at most two, Ago-2 homologs. In contrast to Ago-16 and Ago-30, Ago-68 only shows antiviral activity in case of virus infection, which may suggests its involvement in limiting virus spread by pre-priming yet uninfected cells using systemic RNA silencing. Like mosquitoes, ticks appear to have undergone an expansion of the Piwi clade (Supplementary Figure S5C), though the expansion is smaller and occurred independently, in addition to a possible loss of Ago-3. We show that Dcr-90 is involved in antiviral RNAi against replicon in contrast to Dcr-89 showing significant antiviral activity in case of virus. However, failure of consistent/ efficient knockdown of Dcr-89 between experimental approaches and the borderline increase/significance of Dcr knockdowns on virus infection leaves it open whether or not a second Dcr protein is involved and if there are differences between effects of Dcr knockdowns on replicon and virus. Phylogenetic analysis, dependent on the rooting, maps Dcr-89 in a cluster with Dcr-2 proteins in insects. Dcr-2 is critical for the exogenous antiviral siRNA pathway in *Drosophila*, and presents a limiting factor for sufficient knockdown involving exogenous RNAi (using dsRNA) in this organism (14). Dcr-89 could act in a similar way in ticks, which may explain the lack of consistent knockdown. Dcr-90 showed an antiviral effect in IDE8 cells despite clustering with Dcr-1 proteins which have not yet been reported as antiviral in flies or mosquitoes. It cannot be excluded that potential antiviral functions of some Ago/Dcr proteins we describe here may have been missed due to inefficient knockdown of the transcript; however our results already show that mechanisms in ticks may differ in detail from those present in insects.

A key feature of antiviral RNAi in mosquitoes is the production of 21 nt viRNA molecules (10,11). The majority of viRNAs in IDE8 cells are 22 nt in length [as reported for viRNAs of the positive strand nodavirus in *C. elegans* (45,46)]. The same observation was made if an RNAi response was artificially induced by dsRNA. As the length of the siRNAs or viRNAs is mostly dependent on the Dcr enzyme, this indicates a key difference between *I. scapularis* and insect Dcr proteins. In insects, miRNA molecules differ from siRNA molecules (22 versus 21 nt) as they are mostly produced by Dcr-1. The antiviral effect of Dcr-90, which clusters with insect Dcr-1 proteins, and the production of 22 nt viRNAs points to differences between the antiviral RNAi pathways in *I. scapularis* and insects. Small RNAs of 22 nt were also found to be the major class of small RNA molecules that map to the genome of *I. scapularis*.

Little is known about the dsRNA substrate for Dcr-2 and the origin of viRNAs. Findings by us and others suggest that dsRNA replicative intermediates are Dcr-2 substrates in mosquitoes and derived cell lines and show that hot and cold spots of viRNAs are present along arbovirus genomes/antigenomes (21,38,40–43). This is in agreement with our results for SCRV and transfected dsRNA. In contrast, LGTV viRNAs map at highest frequencies to or around the 5′ and 3′ termini. In contrast, similar regions present in DENV and WNV are not particularly targeted by the RNAi machinery in mosquitoes (41–43). It has to be mentioned that recent work has shown that certain hot and cold spot observations are due to cloning bias of the small RNAs (59,60). The presence of small RNAs mapping to the non-coding strand of SCRV with a similar frequency as to the coding strand, supports the dsRNA genome as inducer molecule even with cloning bias. The same dsRNA-mediated induction may explain the bias of targeting the 5′ and 3′ genome termini of LGTV in IDE8 cells. A previously described replication-incompetent TBEV replicon (C17Fluc NS5 GAA) (23) behaved similar as the corresponding wild-type replicon in IDE8 cells with regards to luciferase production over time [in contrast to BHK where it shows reduction of luciferase production overtime as previously reported (23)] and production/ mapping of TBEV-specific small RNAs (Supplementary Figure S8 and Table 2). This suggests replication of the GAA mutant either by the viral replicase or other enzymes with complementing or replicative activity present in the IDE8 cells. Therefore such a mutant can unfortunately not be used to determine whether the observed TBEV-specific small RNAs are produced from incoming single stranded RNA, dsRNA replication intermediates or partial dsRNA.

Differences in the number of cells targeted by replicon or virus and the amount of virus/replicon RNA per cell could explain the difference in production of overall LGTV-specific small RNAs for replicon versus virus-infected IDE8 cells. Infection by full-length virus may also hide and limit the antiviral RNAi response in IDE8 cells more efficiently than replicon RNA which misses the coding sequences for structural proteins. Besides, the presence of structural proteins and nucleotide sequence (and thus changes in overall length of the viral RNA) may explain the observation that distribution of replicon viRNA versus virus shows some difference. Despite these differences though, LGTV viRNAs share common characteristics (bias for 22 nts viRNAs and targeting areas around the 5′ and 3′ genome termini) which are different to flavivirus-specific viRNAs reported in mosquitoes (41–43).

The detection of LGTV-specific viRNAs indicates the ability of the RNAi response to target the virus, raising the question: how can the virus still replicate in tick cells? Plant and ‘true insect’ viruses encode RSS proteins that interfere with the antiviral RNAi to allow successful viral infection (14,61). No arbovirus RSS protein is known, but an evasion strategy has been suggested for the alphavirus SFV (21) and the sfRNA molecules of mosquito-borne viruses interfere with RNAi responses (22). The 3′UTRs of tick- and mosquito-borne flaviviruses do not share high similarity at the nucleotide level and exchanging these sequences mostly leads to replication-deficient viruses (62–64). Despite this, bioinformatic modeling suggested a highly similar secondary RNA structure profile in the 3′ UTR of arthropod-borne flaviviruses, production and interference with the RNAi response was shown of TBEV and LGTV. WNV sfRNA is believed to mediate RSS activity by acting as a competitive substrate for Dcr (22). In contrast to WNV and DENV UTRs that do not appear to be specifically targeted by Dcr (41–43), the 3′ UTR of the LGTV and TBEV replicon in IDE8 cells appears to be a target for Dcr activities; along with the 5′ UTR it generates the highest frequency of viRNAs. The sfRNA RSS activity probably results in less powerful activity than the known protein-based RSS of insect viruses. Expression of an RSS protein by alphaviruses results in reduced mosquito survival (40,65). Using a weak suppressor such as sfRNA may allow for sufficient levels of replication needed for successful transmission.

Taken together, our findings define details of the tick antiviral RNAi response and its interference by tick-borne arboviruses. They show several important differences in antiviral RNAi between different classes of arbovirus vectors (*Arachnida* versus *Insecta*) and broaden our knowledge about arthropod antiviral RNAi.

## ACCESSION NUMBERS

ERP006219.

## SUPPLEMENTARY DATA

Supplementary Data are available at NAR Online.

SUPPLEMENTARY DATA
