# Supplementary material for: Induction and suppression of tick cell antiviral RNAi responses by tick-borne flaviviruses
Source: Nucleic Acids Res. 2014 Jul 22;42(14):9436–46. doi: 10.1093/nar/gku657 (PMC4132761; doi:10.1093/nar/gku657)
Supplement: SUPPLEMENTARY DATA [file supp_42_14_9436__index.html]

Induction and suppression of tick cell antiviral RNAi responses by tick-borne flaviviruses — Induction and suppression of tick cell antiviral RNAi responses by tick-borne flaviviruses — SUPPLEMENTARY DATA 

# Induction and suppression of tick cell antiviral RNAi responses by tick-borne flaviviruses

## SUPPLEMENTARY DATA

**Files in this Data Supplement:**

- SUPPLEMENTARY DATA
