# Supplementary material for: Induction and suppression of tick cell antiviral RNAi responses by tick-borne flaviviruses
Source: Nucleic Acids Res. 2014 Jul 22;42(14):9436–46. doi: 10.1093/nar/gku657 (PMC4132761; doi:10.1093/nar/gku657)
Supplement: SUPPLEMENTARY DATA [file supp_gku657_nar-03688-v-2013-File009.pdf]

Supplementary Data

Materials and Methods

Construction of LGTV replicon.

This LGTV replicon construct contains the viral 5'UTR, the first 17 residues of the N-terminal region of the capsid protein, corresponding to nucleotides 3789-3810 (numbering according to the full-length sequence of E5), the terminal 27 residues of E protein (nucleotides 6046-6124), followed by the entire nonstructural region (NS1-5) and the 3' UTR. The first 17 residues of the capsid protein were retained since they function as the RNA cyclisation sequence. The terminal 27 residues of E protein were also retained because this region serves as an internal signal sequence for establishing the proper topology of the polyprotein in the ER membrane and targeting of the nonstructural protein NS1 to the secretory pathway (67). The large deletion extending from the beginning of the prM gene through most of the region coding for the E protein was introduced into the E5 cDNA sequence using standard overlapping PCR techniques (details available upon request). The deleted region was replaced by unique restriction sites *Sna*BI and *Xho*I (to facilitate construction of the E5 replicon), followed by the recognition sequence for the LGTV protease NS2B/3. This construct was designated E5 2B/3. To construct the E5repRluc2B/3 replicon, the *Renilla* luciferase (Rluc) gene was amplified by PCR from the yellow fever virus (YFV) replicon YFV Rluc 2A (68) using forward and reverse primers that incorporated a *Sna*BI and an *Xho*I restriction site respectively. This amplified product was then cloned downstream of the

capsid protein sequence, into the *Sna*BI and *Xho*I restriction sites of the E5 2B/3 construct. The resulting cDNA replicon construct was designated E5repRluc2B/3.

Immunostaining and production of fluorescently labelled nucleic acids

dsRNA or LGTV E5 rep2B/3 Rluc was *in vitro* transcribed as previously described (see materials and methods), but this time in the presence of fluorescin-labelled UTP (Roche). Commercial fluorescin-labelled plasmid DNA was also used (Mirus). Transfected/infected IDE8 cells were fixed by formaldehyde and immunostaining of infected cells was performed mainly as previously described (28). In short cells were permeabilised by 0.3% Triton/PBS for 30 min, blocked with CAS-Block and infected cells were stained with flavivirus Envelope-specific antibody (1:100) (Millipore) or LGTV specific NS3 antibody (1:100) (69) and anti-mouse antibody conjugated with AlexaFluor594 (1:1000) or anti-chicken antibody conjugated with AlexaFluor594 (1:1000). Fluorescence was detected on a Zeiss LSM Meta microscope.

TBEV replicon: in vitro transcription, time course and Illumina sequencing

The TBEV replicons (wt and non-replicating GAA mutant) was previously described as C17Fluc or C17Fluc NS5-GAA, respectively (23). C17Fluc or C17Fluc NS5-GAA was linearised by NheI and *in vitro* transcribed in the presence of cap analogue using a T7 Megascript kit (Ambion) according to the manufacturer`s protocol. *In vitro* transcribed RNA was transfected into IDE8 or BHK cells, lysed at different time points and luciferase measured. For small RNA sequencing, RNA was isolated at 24 hpt, Illumina sequencing and bioinformatics analysis performed as described in the Materials and methods.

67. Lindenbach, B.D., Pragai, B.M., Montserret, R., Beran, R.K., Pyle, A.M., Penin, F. and Rice, C.M. (2007) The C terminus of hepatitis C virus NS4A encodes an electrostatic switch that regulates NS5A hyperphosphorylation and viral replication. J Virol, 81, 8905-8918.

68. Jones, C.T., Patkar, C.G. and Kuhn, R.J. (2005) Construction and applications of yellow fever virus replicons. Virology, 331, 247-259.

69. Taylor, R.T., Lubick, K.J., Robertson, S.J., Broughton, J.P., Bloom, M.E., Bresnahan, W.A. and Best, S.M. (2011) TRIM79alpha, an interferon-stimulated gene product, restricts tick-borne encephalitis virus replication by degrading the viral RNA polymerase. Cell Host Microbe, 10, 185-196.

Supplementary Figures

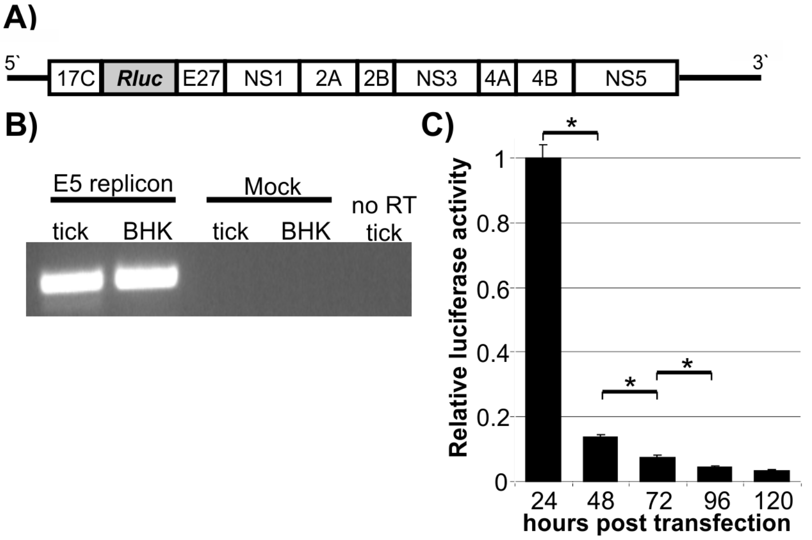

Figure S1: Langat virus (LGTV) replication in *I. scapularis*-derived IDE8 cells.

(A) Schematic presentation of the LGTV strain E5 derived replicon expressing *Renilla* luciferase (Rluc): E5repRluc 2B/3. **(B)** IDE8 and BHK cells were transfected with mock DNA or *in vitro*-transcribed E5rep Rluc 2B/3 RNA. RNA was isolated 24 hpt, reverse transcribed with antigenome specific primers and PCR products were run on a gel. **(C)** IDE8 cells were transfected with *in vitro*-transcribed E5repRluc2B/3 RNA, and *Renilla* luciferase (Rluc) expression was measured at 24, 48, 72, 96 and 120 hpt. The mean with standard error is shown for three independent experiments performed in duplicate. The luciferase expression level measured at 24 hpt was set at 1.0. * indicate significance by Tukey`s HSD (p≤0.05).

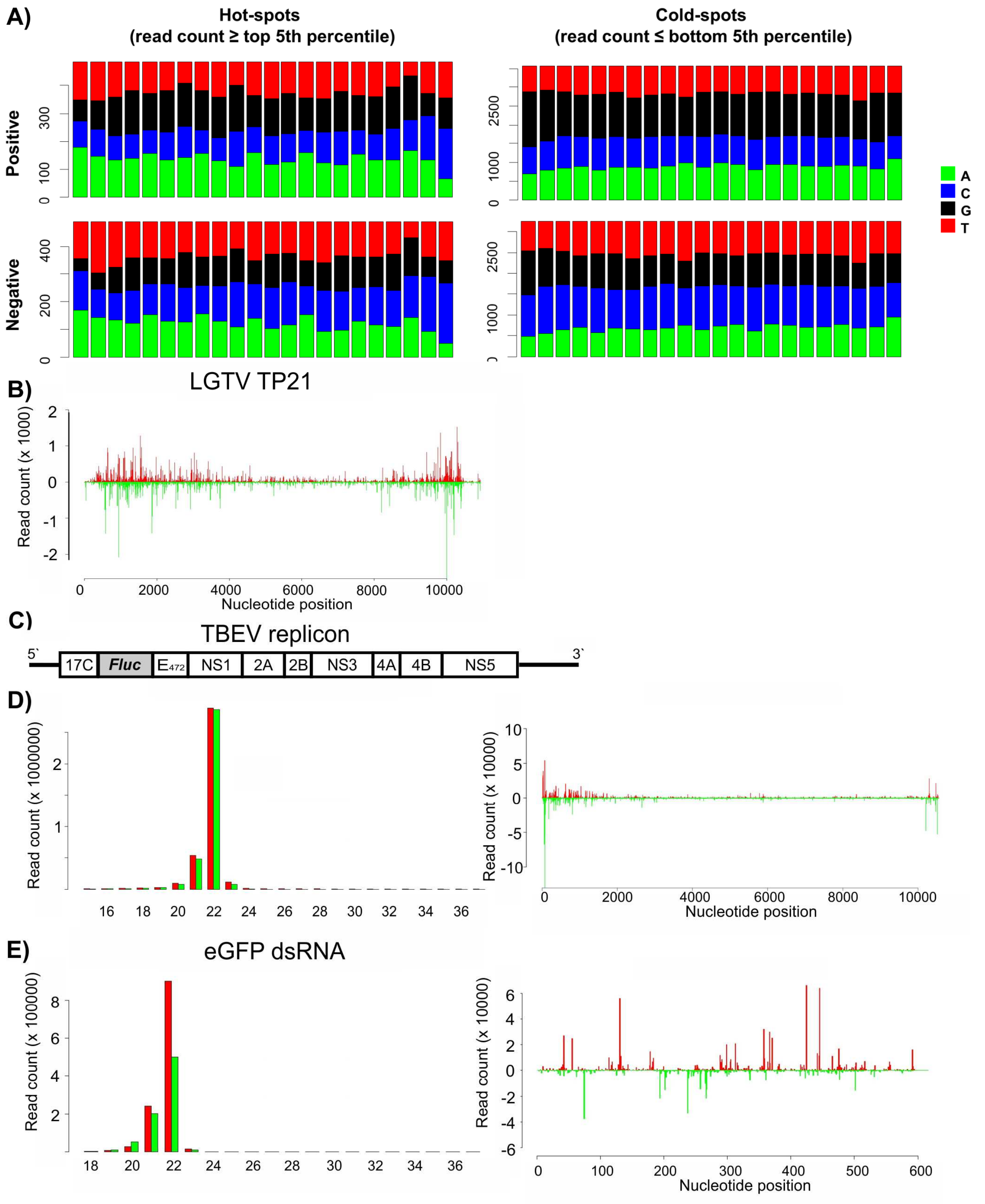

Figure S2: Characteristics of LGTV specific 22 nt small RNAs in IDE8 cells.

(A) Base composition of LGTV specific 22 nts small RNAs of hot spots (≥ reads than top 5% of all 22-mer locations) versus cold spots (≤ reads than bottom 5% of all 22-mer locations). **(B)** Frequency distribution of 22 nt small RNA molecules mapping to LGTV TP 21 in IDE8 cells.

“Zoom in” of Fig. 1b right panel. **(C)** Schematic representation of the TBEV C17Fluc replicon expressing Firefly luciferase (Fluc). **(D, E)** Size distribution of small RNA molecules (left panel) and frequency distribution of 22 nt small RNA molecules (right panel) mapping to 720 nt eGFP sequence specific dsRNA **(E)** or TBEV C17Fluc replicon **(D)** in IDE8 cells. Red, small RNAs map to the coding strand/genome; green, small RNAs to the non-coding strand/antigenome. The y-axis shows the frequency of the 22 nt siRNAs mapping to the corresponding nucleotide position on the x-axis. Positive numbers (red peaks) represent the frequency of siRNAs mapping to the coding strand/ genome (in 5’-3’ orientation) and green peaks(negative numbers) siRNAs mapping to the non-coding strand/antigenome (in 3’-5’ orientation).

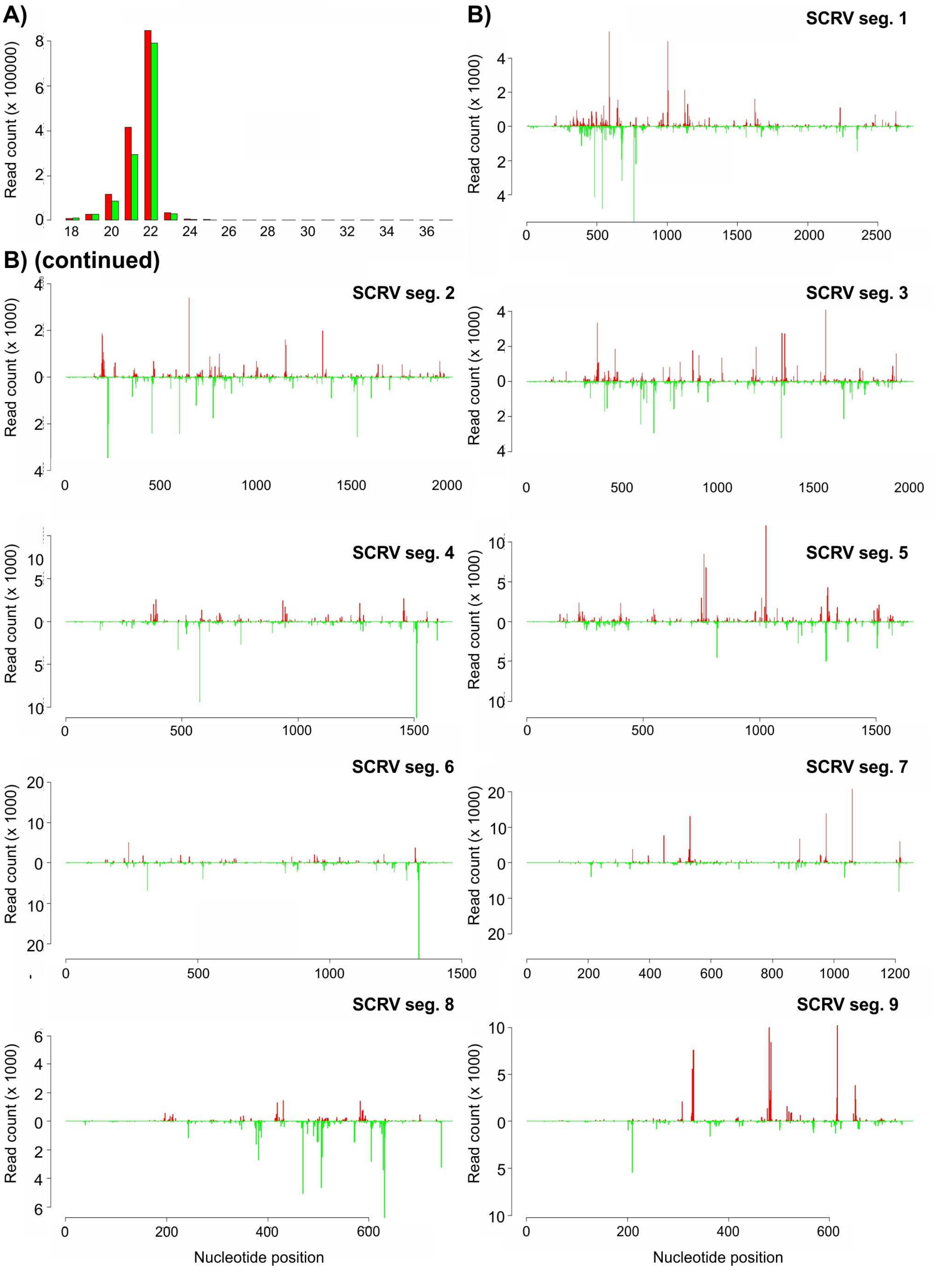

Figure S3: Characterisation of St. Croix River virus (SCRV) viRNAs in IDE8 cells.

(A) Size distribution of small RNA molecules mapping to the SCRV genome in persistently-

infected IDE8 cells. Red small RNAs map to the coding strand and green small RNAs to the non-coding strand of the dsRNA genome. **(B)** Frequency distribution of 22 nt small RNA molecules mapped to the segments of SCRV. The y-axis shows the frequency of the 22 nt siRNAs mapping to the corresponding nucleotide position on the x-axis. Positive numbers and red peaks represent the frequency of viRNAs mapping to the coding strand (in 5’-3’ orientation) and green peaks/ negative numbers viRNAs mapping to the non-coding strand (in 3’-5’ orientation).

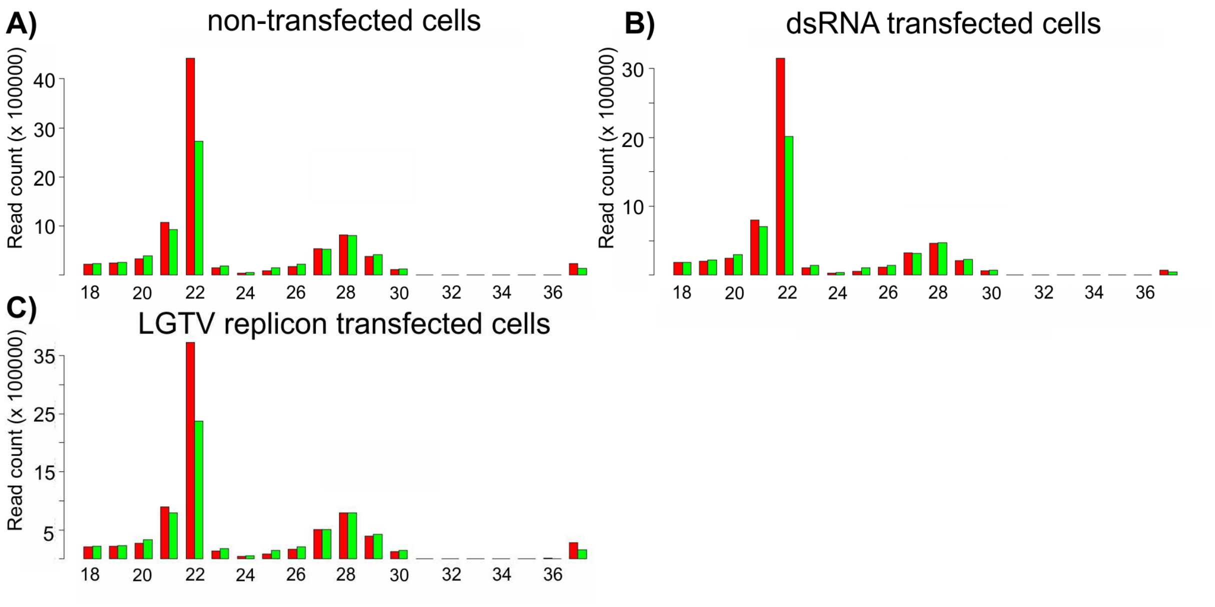

Figure S4: Characterisation of endogenously-derived small RNAs in IDE8 cells.

Size distribution of small RNA molecules mapping to the *I. scapularis* genome in IDE8 cells either **(A)** non-treated, **(B)** transfected with 720 nt eGFP sequence-specific dsRNA or **(C)** transfected with LGTV E5repRluc2B/3 replicon. Red maps to one of the chromosomal strands and green to the other. See also Fig. S2.

A)

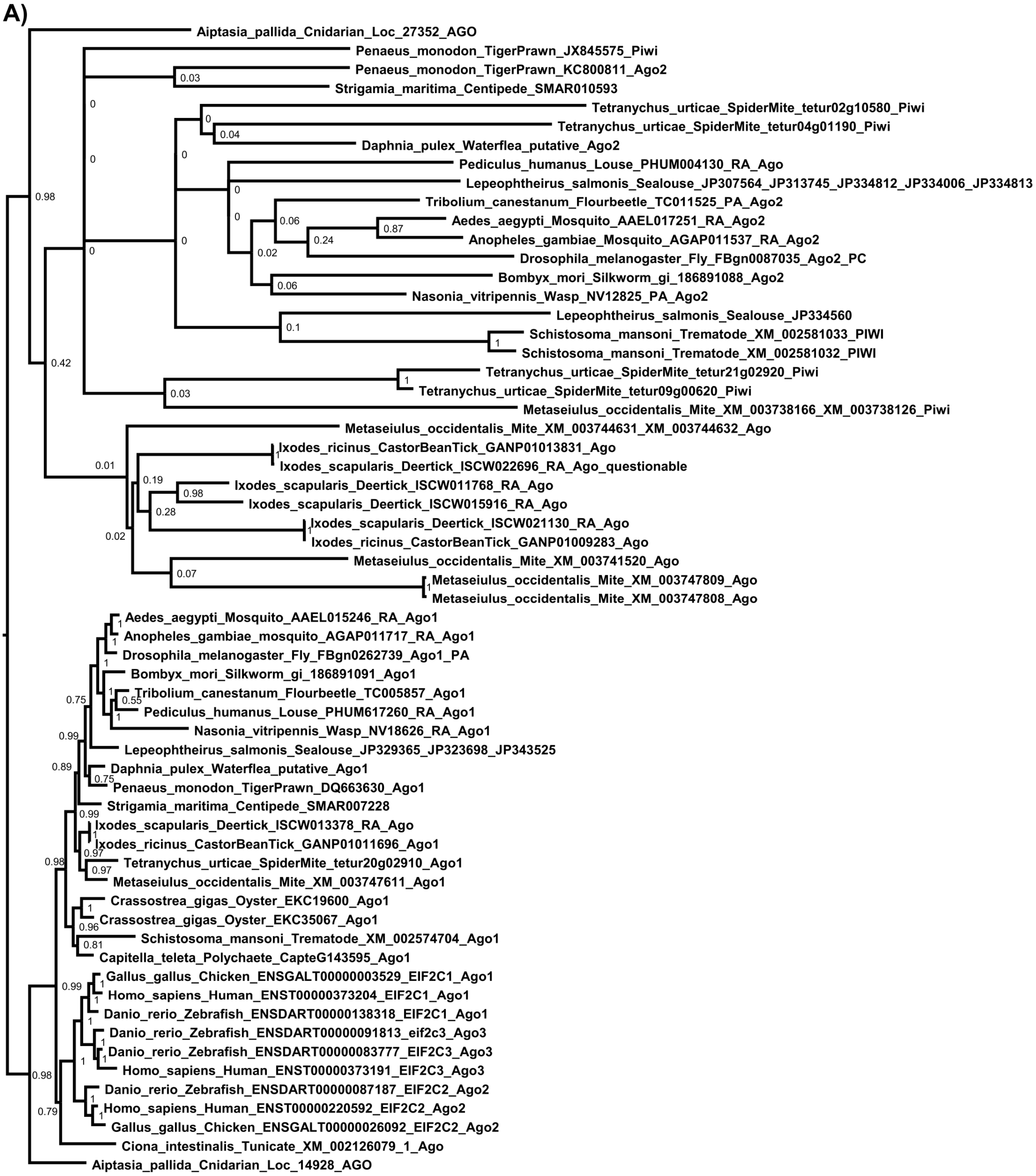

Aiptasia_pallida_Cnidarian_Loc_27352_AGO
Penaeus_monodon_TigerPrawn_JX845575_Piwi
Penaeus_monodon_TigerPrawn_KC800811_Ago2
Strigamia_maritima_Centipede_SMAR010593
Tetranychus_urticae_SpiderMite_tetur02g10580_Piwi
Tetranychus_urticae_SpiderMite_tetur04g01190_Piwi
Daphnia_pulex_Waterflea_putative_Ago2
Pediculus_humanus_Louse_PHUM004130_RA_Ago
Lepeophtheirus_salmonis_Sealouse_JP307564_JP313745_JP334812_JP334006_JP334813
Tribolium_canestanum_Flourbeetle_TC011525_PA_Ago2
Aedes_aegypti_Mosquito_AAEL017251_RA_Ago2
Anopheles_gambiae_Mosquito_AGAP011537_RA_Ago2
Drosophila_melanogaster_Fly_FBgn0087035_Ago2_PC
Bombyx_mori_Silkworm_gi_186891088_Ago2
Nasonia_vitripennis_Wasp_NV12825_PA_Ago2
Lepeophtheirus_salmonis_Sealouse_JP334560
Schistosoma_mansoni_Trematode_XM_002581033_PIWI
Schistosoma_mansoni_Trematode_XM_002581032_PIWI
Tetranychus_urticae_SpiderMite_tetur21g02920_Piwi
Tetranychus_urticae_SpiderMite_tetur09g00620_Piwi
Metaseiulus_occidentalis_Mite_XM_003738166_XM_003738126_Piwi
Metaseiulus_occidentalis_Mite_XM_003744631_XM_003744632_Ago
Ixodes_ricinus_CastorBeanTick_GANP01013831_Ago
Ixodes_scapularis_Deertick_ISCW022696_RA_Ago_questionable
Ixodes_scapularis_Deertick_ISCW011768_RA_Ago
Ixodes_scapularis_Deertick_ISCW015916_RA_Ago
Ixodes_scapularis_Deertick_ISCW021130_RA_Ago
Ixodes_ricinus_CastorBeanTick_GANP01009283_Ago
Metaseiulus_occidentalis_Mite_XM_003741520_Ago
Metaseiulus_occidentalis_Mite_XM_003747809_Ago
Metaseiulus_occidentalis_Mite_XM_003747808_Ago
Aedes_aegypti_Mosquito_AAEL015246_RA_Ago1
Anopheles_gambiae_mosquito_AGAP011717_RA_Ago1
Drosophila_melanogaster_Fly_FBgn0262739_Ago1_PA
Bombyx_mori_Silkworm_gi_186891091_Ago1
Tribolium_canestanum_Flourbeetle_TC005857_Ago1
Pediculus_humanus_Louse_PHUM617260_RA_Ago1
Nasonia_vitripennis_Wasp_NV18626_RA_Ago1
Lepeophtheirus_salmonis_Sealouse_JP329365_JP323698_JP343525
Daphnia_pulex_Waterflea_putative_Ago1
Penaeus_monodon_TigerPrawn_DQ663630_Ago1
Strigamia_maritima_Centipede_SMAR007228
Ixodes_scapularis_Deertick_ISCW013378_RA_Ago
Ixodes_ricinus_CastorBeanTick_GANP01011696_Ago1
Tetranychus_urticae_SpiderMite_tetur20g02910_Ago1
Metaseiulus_occidentalis_Mite_XM_003747611_Ago1
Crassostrea_gigas_Oyster_EKC19600_Ago1
Crassostrea_gigas_Oyster_EKC35067_Ago1
Schistosoma_mansoni_Trematode_XM_002574704_Ago1
Capitella_teleta_Polychaete_CapteG143595_Ago1
Gallus_gallus_Chicken_ENSGALT00000003529_EIF2C1_Ago1
Homo_sapiens_Human_ENST00000373204_EIF2C1_Ago1
Danio_rerio_Zebrafish_ENSDART00000138318_EIF2C1_Ago1
Danio_rerio_Zebrafish_ENSDART00000091813_eif2c3_Ago3
Danio_rerio_Zebrafish_ENSDART00000083777_EIF2C3_Ago3
Homo_sapiens_Human_ENST00000373191_EIF2C3_Ago3
Danio_rerio_Zebrafish_ENSDART00000087187_EIF2C2_Ago2
Homo_sapiens_Human_ENST00000220592_EIF2C2_Ago2
Gallus_gallus_Chicken_ENSGALT00000026092_EIF2C2_Ago2
Ciona_intestinalis_Tunicate_XM_002126079_1_Ago
Aiptasia_pallida_Cnidarian_Loc_14928_AGO

B)

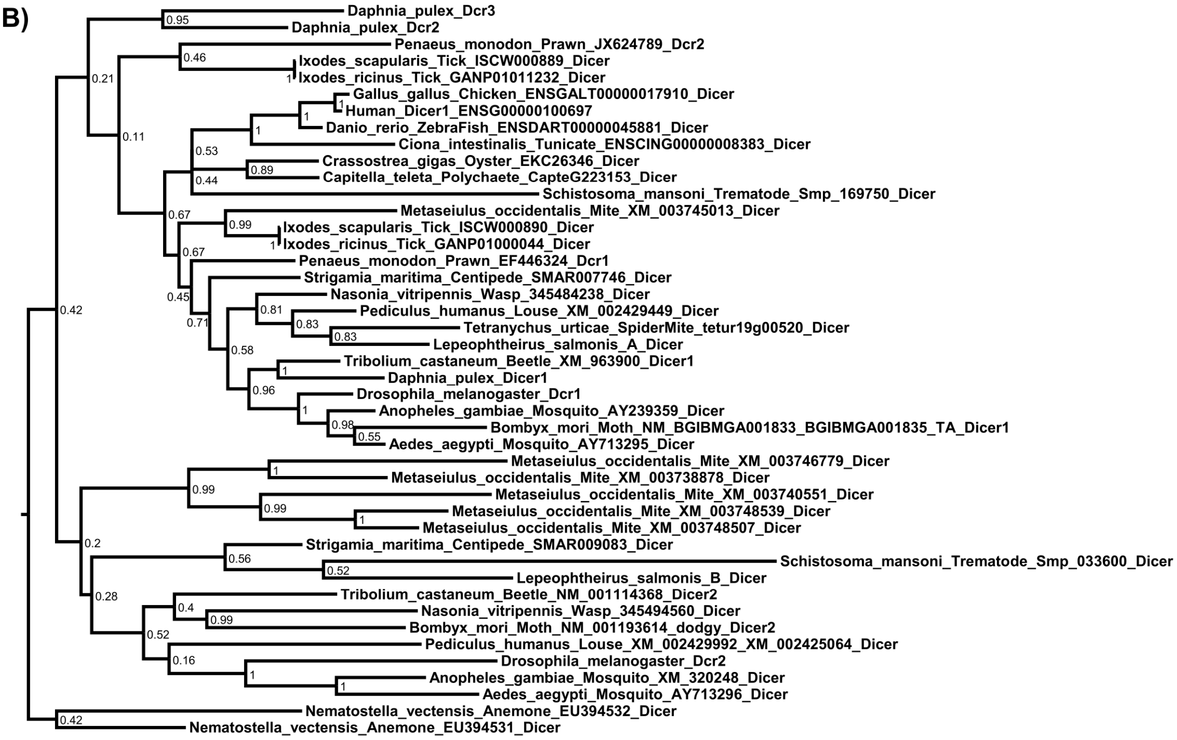

C)

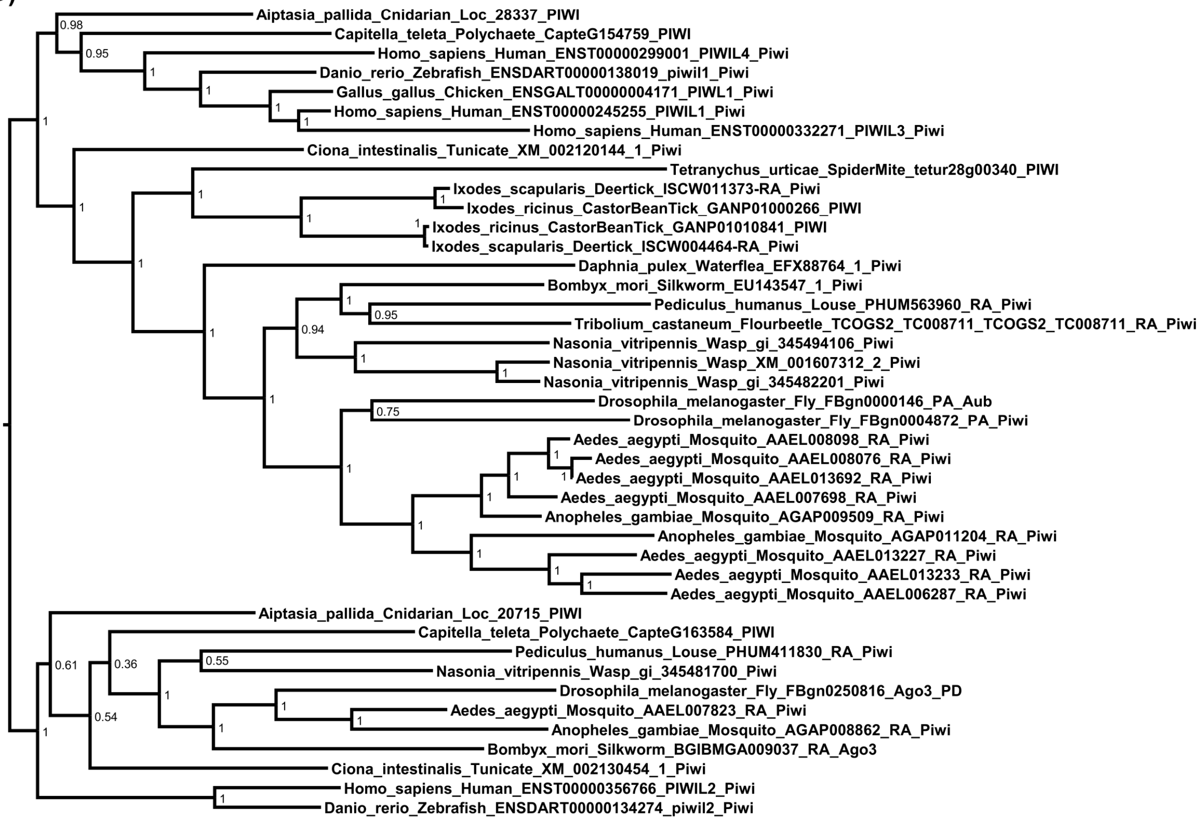

Figure S5: Phylogenetic tree of genes encoding Ago, Dcr or Piwi proteins using the Bayesian approach and full name labelling.

Gene tree for metazoan Ago-subfamily **(A),** Dcr-subfamily **(B)** or Piwi-subfamily (**C**) genes constructed using a Bayesian approach under a GTR model (nodes are labelled if they receive >90% support; see Experimental Procedures). Panel A and B are identical to Fig. 2, except that all nodes are labelled, and sequences are labelled with their full species names and Genbank or Genome identifiers.

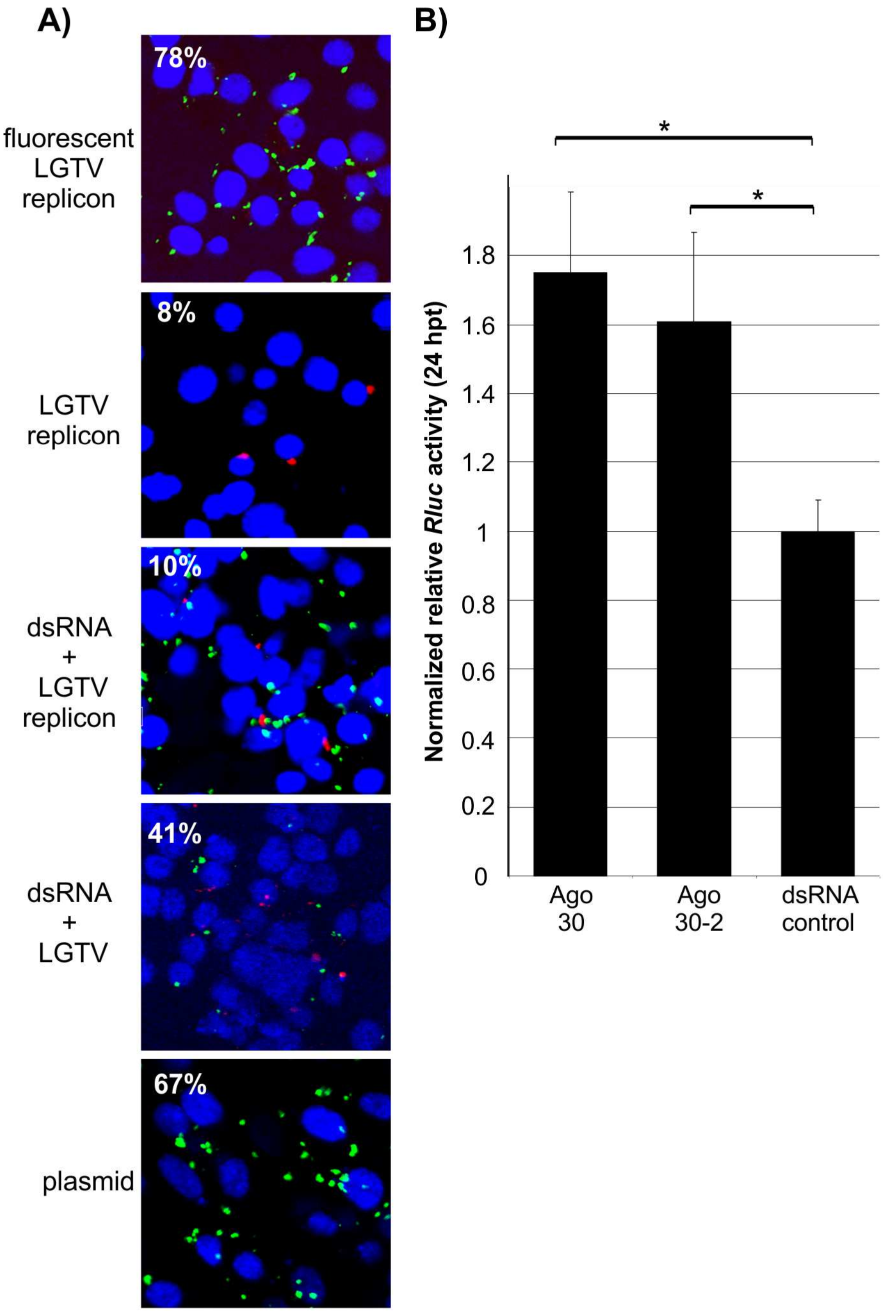

Figure S6: Characterization of IDE8 cells.

(A) Detection of transfected fluorescently labelled LGTV replicon RNA or plasmid in IDE8 cells as well as fluorescently labelled dsRNA (green signals), where indicated in combination with LGTV infection or LGTV replicon transfection as monitored by immunofluorescence detection of either the Envelope protein (virus) or NS3 (replicon), respectively (red signals).

Cell nuclei were stained by DAPI (blue). Percentage of cells transfected or transfected/infected is indicated. **(B)** IDE8 cells treated with dsRNA specific to two different regions of Ago-30 (Ago30 or Ago30-2) or eGFP (control) were transfected with capped *in vitro*- transcribed LGTV E5repRluc2B/3 replicon RNA, and Rluc activity was determined at 24 hpt. The mean of two independent experiments performed in triplicate with standard error is shown; * represent $p<0.05$, Student t-test.

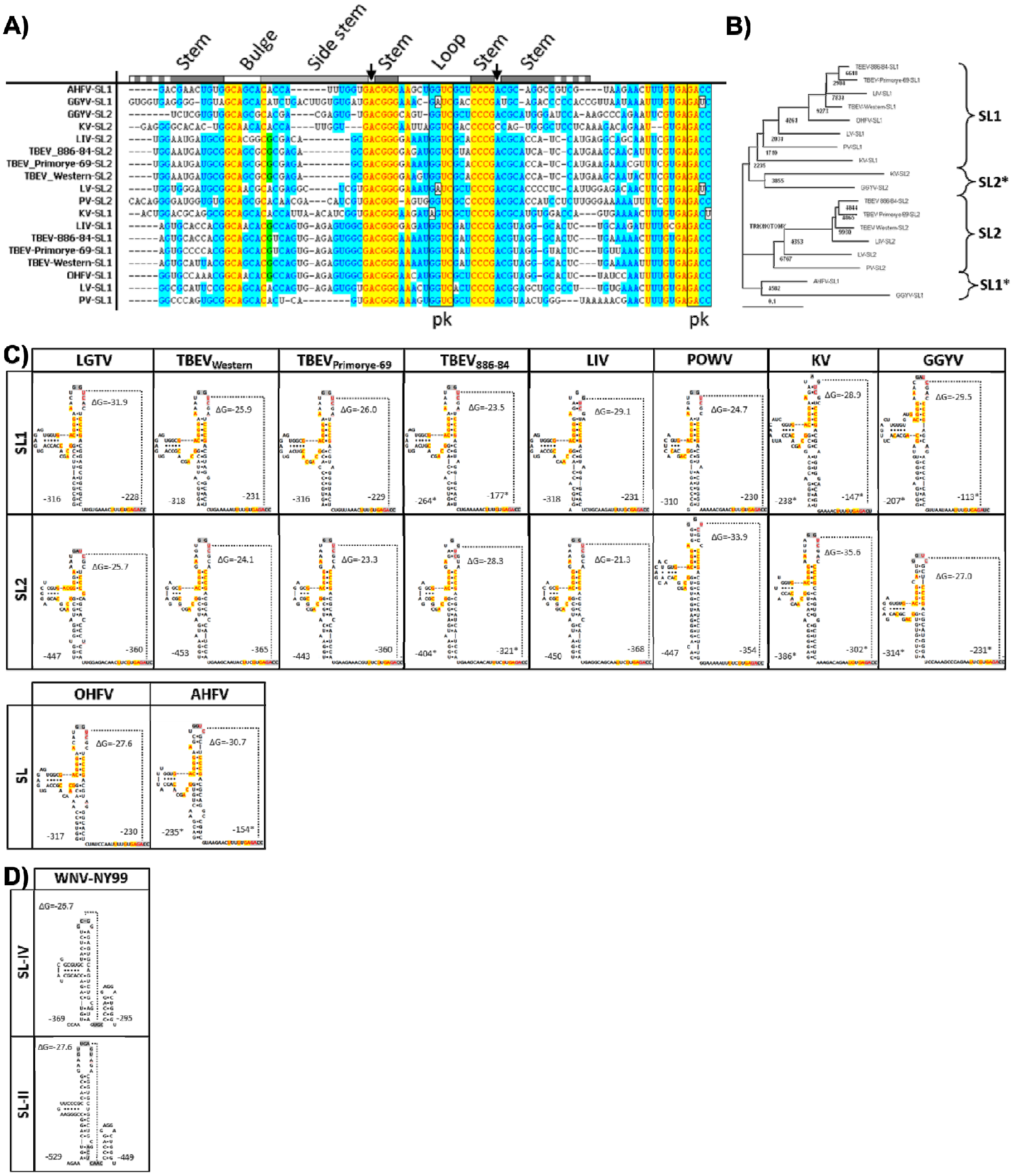

Figure S7: Analysis of subgenomic flavivirus RNA in the 3`UTR of tick-borne flaviviruses.

(A) Clustal alignment of several 3`UTR of tick-borne flaviviruses. LGTV: Langat virus (NC_003690.1), TBEV: tick-borne encephalitis virus (NC_001672.1, EF469662.1, EU816453.1), LIV: louping ill virus (NC_001809.1), POWV: Powassan virus (NC_003687.1), KV: Karshi virus

(NC_006947.1), GGYV: Gadgets Gully virus (no Genbank file available), OHFV: Omsk hemorrhagic fever virus (NC_005062.1), AHFV: Alkhurma hemorrhagic fever virus (NC_004355.1). SL1 and SL2 regions present in their 3`UTR; the structural aspects of the structures are indicated on top of the alignment. The base stem is indicated in dark grey, the side stemloop is indicated in a lighter grey. Both the bulge before the side stemloop and the loop on top of the structure are indicated in white. The pseudoknot is indicated with black borders in the alignment itself, as are individual nucleotides that represent compensatory mutations. The two conserved unpaired nucleotides are indicated with an arrow. **(B)** A phylogram of the two SL regions is shown, generated by Neighbor-joining with 10,000 bootstraps. On the nodes the bootstrap values are depicted. Two SL2 structures that group closer to the clade of SL1 structures and SL1 structures that could not be grouped with either the SL1 or SL2 clade are indicated with an * behind the brackets. **(C)** The predicted structure of either both or only one SL structure of tick-borne flaviviruses is depicted. The compensatory nucleotide mutations in the pseudoknot region are indicated by arrows. **(D)** Same as **(C)** for West Nile virus New York 99 strain.

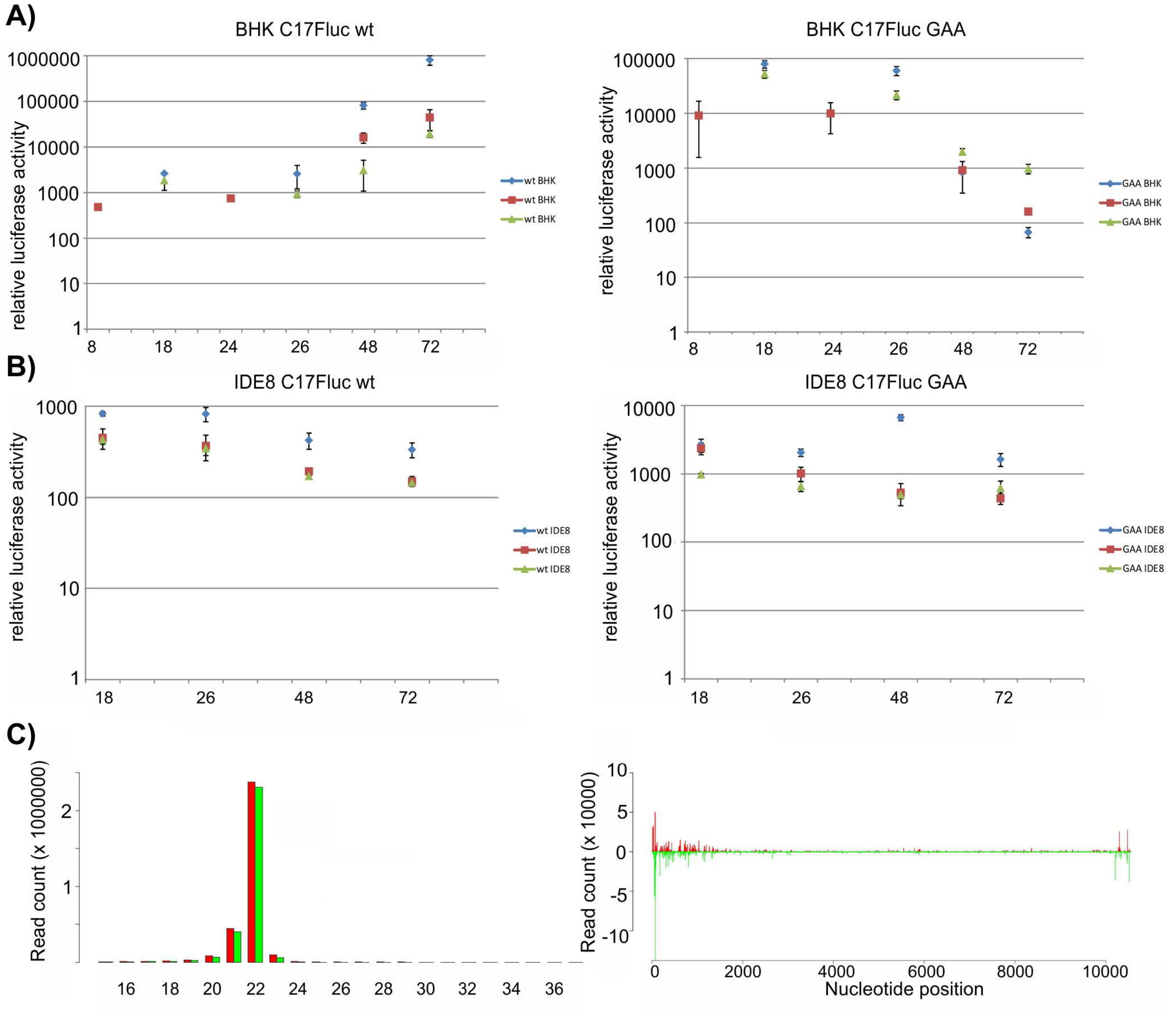

Figure S8: Characterization of the TBEV replicon NS5-GAA mutant.

Firefly luciferase (Fluc) expression was measured at different hpt of BHK-21 **(A)** or IDE8 **(B)** cells transfected with *in vitro*-transcribed TBEV replicon RNA, either C17 Fluc wt (left panel) or C17Fluc GAA mutant (right panel). The mean with standard error is shown for three independent experiments performed in triplicate. **(C)** Size distribution of small RNA molecules mapping to TBEV C17Fluc GAA mutant replicon at 48 hpt (left panel) and frequency distribution of 22 nt small RNA molecules mapped to the TBEV C17Fluc GAA (right panel). The y-axis shows the frequency of the 22 nt siRNAs mapping to the corresponding

nucleotide position in the x-axis. Positive numbers and read peaks represent the frequency of viRNAs mapping to the genome (in 5’-3’ orientation) and green peaks/negative numbers to the antigenome (in 3’-5’ orientation).
